# Comparative Transcriptome Profiling Reveals Potential Candidate Genes, Transcription Factors, and Biosynthetic Pathways for Phosphite Response in Potato (*Solanum tuberosum* L.)

**DOI:** 10.3390/genes13081379

**Published:** 2022-08-01

**Authors:** Richard Dormatey, Tianyuan Qin, Yihao Wang, Benjamin Karikari, Simon Dontoro Dekomah, Youfang Fan, Zhenzhen Bi, Panfeng Yao, Kazim Ali, Chao Sun, Jiangping Bai

**Affiliations:** 1 Department of Crop Genetics and Breeding, College of Agronomy, Gansu Agricultural University, Lanzhou 730070, China; rmddormatey@gmail.com (R.D.); qty1637835362@sina.com (T.Q.); xiaohao9639@163.com (Y.W.); dekomahsimon42@gmail.com (S.D.D.); fanyf3@163.com (Y.F.); bizz@gsau.edu.cn (Z.B.); kazim76@gmail.com (K.A.); 2 State Key Laboratory of Aridland Crop Science, Gansu Agricultural University, Lanzhou 730070, China; yaopf@gsau.edu.cn; 3 CSIR-Crops Research Institute, P.O. Box 3785, Kumasi 00233, Ghana; 4 Department of Crop Science, Faculty of Agriculture, Food and Consumer Sciences, University for Development Studies, Tamale 00233, Ghana; benkarikari1@gmail.com; 5 National Institute for Genomics and Advanced Biotechnology, National Agricultural Research Centre, Park Road, Islamabad 45500, Pakistan

**Keywords:** phosphite stress, potato, transcriptome, phytohormones, transcription factors, photosynthesis

## Abstract

The study was conducted with C31 and C80 genotypes of the potato (*Solanum tuberosum* L.), which are tolerant and susceptible to phosphite (Phi, H_2_PO_3_), respectively. To decipher the molecular mechanisms underlying tolerance and susceptibility to Phi in the potato, RNA sequencing was used to study the global transcriptional patterns of the two genotypes. Media were prepared with 0.25 and 0.50 mM Phi, No-phosphorus (P), and 1.25 mM (phosphate, Pi as control). The values of fragments per kilobase of exon per million mapped fragments of the samples were also subjected to a principal component analysis, grouping the biological replicates of each sample. Using stringent criteria, a minimum of 819 differential (DEGs) were detected in both C80-Phi-0.25_vs_C80-Phi-0.50 (comprising 517 upregulated and 302 downregulated) and C80-Phi-0.50_vs_C80-Phi-0.25 (comprising 302 upregulated and 517 downregulated) and a maximum of 5214 DEGs in both C31-Con_vs_C31-Phi-0.25 (comprising 1947 upregulated and 3267 downregulated) and C31-Phi-0.25_vs_C31-Con (comprising 3267 upregulated and 1947 downregulated). DEGs related to the ribosome, plant hormone signal transduction, photosynthesis, and plant–pathogen interaction performed important functions under Phi stress, as shown by the Kyoto Encyclopedia of Genes and Genomes annotation. The expressions of transcription factors increased significantly in C31 compared with C80. For example, the expressions of *Soltu.DM.01G047240*, *Soltu.DM.08G015900*, *Soltu.DM.06G012130*, and *Soltu.DM.08G012710* increased under P deficiency conditions (Phi-0.25, Phi-0.50, and No-P) relative to the control (P sufficiency) in C31. This study adds to the growing body of transcriptome data on Phi stress and provides important clues to the Phi tolerance response of the C31 genotype.

## 1. Introduction

Phosphorus (P) is an essential mineral for the formation and proliferation of cells. P is a component of phospholipids and nucleic acids as well as a source of energy and a signal transducer [1]. P is taken up by plant roots mainly in the form of inorganic phosphate (HPO_4_^2–^ and H_2_PO_4_^–^). However, because Pi is readily aggregated with cations or transformed to its organic state, its accessibility in the soil is generally limited [2]. To promote the utilization of Pi from the soil, plants have evolved a series of adaptations called Pi starvation responses (PSRs) that alter the distribution and utilization of Pi in plants in response to low Pi availability. PSRs have been associated with changes in root system architecture, increased expression of Pi transporters, release of phosphatases and RNases, modification of membrane lipids, buildup of anthocyanins, and rapid energy production [3]. Several studies have addressed the transcriptional control of PSRs. When Pi was limited in the growth medium, a variety of genes (500–2000) were upregulated, depending on the experimental design [4,5,6,7].

In the absence of Pi, plants show signs of deficiency, such as increased root growth and a higher ratio of roots to shoots. During this time, the plant’s ability to take up Pi also improves. Molecular analysis of Pi deficiency has revealed evidence of coherent gene expression involving Pi transporters [2,8]. Pi transporters are involved in the extraction of Pi against a concentration gradient through an energy-mediated proton cotransport process [9]. Arsenate, vanadate, and phosphite are examples of ions known to be transported by them [10]. Phi (H_3_PO_3_^–^), commonly known as phosphoric acid or phosphonate, is a Pi-anion isostere in which one of the oxygen atoms associated with the P atom has been replaced by hydrogen. According to Carswell et al. [11], the alkali metal salts of phosphorous acid are referred to as Phi. Phi is touted as an excellent source of P and is often used as a fungicide [10,12,13]. Phi is rapidly taken up by the plant and translocated to its interior [10]. Uptake is pH-dependent and competes with Pi [12]. There is also a similarity between the mobility of Phi and Pi in the xylem and phloem [12]. Despite its comparable structure and mobility, Phi is a non-metabolizable version of Pi that plants cannot use as a major source of P [11,14,15]. The main difference between Pi and Phi is that Pi can be easily converted into organic P molecules immediately after absorption, which is not possible with Phi [10,13]. The transfer of Pi groups is caused by certain enzymes, which can also provide a clue to the difference between Pi and Phi [10]. Bacteria that oxidize Phi to Pi are most likely responsible for their nutritional benefits [16]. Because of this biological transformation, Phi is an important component of the global P cycle, although it does not provide a direct source of nutrients to plants.

Several researchers have found evidence of the deleterious effects of Phi on plant growth and development [11,15,17,18]. Application of Phi to roots of *Allium cepa* and *Brassica nigra* reduced root growth [11,14]. In *Arabidopsis*, root development, elongation, and root hair formation were induced [15]. Phi induced decreased activity of nucleolytic enzymes and Pi deficiency induced gene expression in *Arabidopsis* [15]. According to Ticconi et al. [15], the adverse effects of Phi could be attenuated by an additional supply of Pi. Since Phi suppresses several responses triggered by Pi deficiency, it has been hypothesized that the signal transduction patterns leading to Pi deficiency responses have been damaged [11]. To support the development of a better use of Phi in modern agriculture, a better knowledge of the underlying molecular signaling pathways associated with the indirect mode of action of Phi is required. 

RNA-sequencing (RNA-seq) is a powerful analytical tool for deciphering molecular mechanisms, structural genes and transcription factors associated with plants under biotic and abiotic stresses [19,20]. Due to its proven approach and rapid development, the cost of data acquisition is reduced and complicated phenomena of gene expression and regulation are revealed [17,18]. This technique has enabled scientists to study the genetic responses of different plant species to biological and abiotic factors, such as drought, high or low temperatures, salt stress, phosphate and nitrogen deficiencies, and pathogen infection [21]. Chickpea genotypes that are salt- or drought-tolerant were studied using RNA-seq under both normal and salt or drought stress situations in asexual and generic plant development [22]. Using RNA-seq technology, leaves of Pi-deficient potatoes were found to have increased protein recycling and altered amino acid production [23,24,25,26]. 

A previous report also indicated that a number of genes annotated with protein fate terms in Gene Ontology (GO) were elevated in the potato with insufficient Pi [23]. According to Wu et al. [24] and Misson et al. [4], Arabidopsis plant leaves respond to Pi deficiency with increased expression of protein degradation genes and inhibition of protein production genes. In addition, eight *pht1* homologous genes were detected in barley under limited Pi [27]. Among the various PHT1 genes, *HvPHT1*;*6* genes were found to transfer phosphorus for expression in the shoot and root [28], and a mycorrhiza-specific gene, *HvPHT1*;*8*, was discovered. The promoters of *HvPht1*;*1* and *HvPht1*;*2* were strongly expressed in barley under P deficiency [29]. Moreover, the RNA-seq tool was used under biotic stress conditions. For example, Huang and colleagues found that pathogenesis-related proteins and salicylic acid genes were significantly less expressed in susceptible potato genotypes compared to the control, indicating higher resistance to pathogens (*Phytophthora infestans*) after Phi treatment [30]. In addition, important ethylene pathway genes, such as *ERF1/2*, jasmonic acid (*JAZ*), and SA (*TGA* and *PR1*) were strongly expressed in Phi-treated seedlings [31]. RNA-seq revealed that the *ACO1* gene was more highly-expressed in Phi-treated potato seedlings than in control plants [32,33], indicating resistance to the pathogen (*P. infestans*). Overexpression of *OsPHR2* leads to a response to phosphate deficiency, suggesting that this gene plays a conserved role in the control of Pi signaling in rice [34]. 

Three other TFs reported to be involved in the control of plant adaptation to Pi deficiency [35,36,37] are *OsPTF1*/*ZmPTF1*, *WRKY75*, and *ZAT6* (zinc finger of Arabidopsis6, a cysteine-2/histidine-2 zinc finger protein). RNA-Seq technology has indeed led to an explosion of genomic and transcriptomic resources in plant sciences. It is a rapid and simple technique to study global gene expression patterns in a variety of situations [38]. To elucidate the underlying molecular mechanisms of Phi tolerance in plants, we performed transcriptome profiling of two contrasting potato (*S. tuberosum* L.) genotypes under 0, 0.25, and 0.50 mM Phi, and No-P. The aim of the present study was to: (i) Identify potential candidate genes for tolerance or susceptibility of potato genotypes to P treatment. (ii) Identify key transcription factors involved in the potato’s response to P treatment, and (iii) investigate key biosynthetic pathways involved in the potato’s response to P treatment. In this study, the analysis of the expression of TFs revealed that relevant gene families, such as WRKY, MYB, NAC, and bHLH, are associated with the Phi-tolerant genotype C31, whereas MAD is associated with the susceptible genotype C80. The expression of key genes underlying the P response in the potato was also investigated. The results of the present study provide the basis for manipulating and understanding the response to phosphite in the potato to exploit its potential for crop production and will facilitate the breeding of Phi-tolerant cultivars. In addition, potential candidate genes could be useful for future functional validation through editing technology and gene overexpression, among others.

## 2. Materials and Methods

### 2.1. Planting Materials and In Vitro Propagation Conditions

The present study was designed to determine whether the differential gene expression profiles of tolerant and susceptible potato genotypes correlate with the phenotypic response to Phi stress and which genes might contribute to this effect. Two potato genotypes (C31 and C80) were used that were classified as moderately tolerant and susceptible based on data from the initial screening for Phi tolerance, respectively. The genotypes were characterized based on physiological traits (number of roots, plant height, root length, and shoot length) under different treatment concentrations (Con, No-P, Phi0.25, and Phi0.50 mM KH_2_PO_3_) in vitro culture. The experiment was conducted in the growth chamber of the Tissue Culture Laboratory of the College of Agronomy, Gansu Agricultural University Lanzhou, China (36°03′ N; 103°40′ E). 

To obtain healthy-grown seedlings for the next experiment, uniform stem explants consisting of cuttings with one node were maintained in vitro under aseptic conditions and propagated on Murashige and Skoog [39] growth medium containing 30 gL^−1^ sucrose and 5 g L^−1^ agar. The pH was raised to 5.8, and the cultures were autoclaved at 121 °C and 15 1b psi for 25 min. The culture was maintained under aseptic conditions in a culture storage chamber at an active temperature of 25 ± 1 °C during the day, 16 h of light and night, 8 h of darkness, an active photosynthetic radiation of 45 mol photons m^−2^ s^−1^, and relative humidity of 55–66% for a growth period of 30 days. After this period, healthy plantlets were selected and subcultured in the MS medium supplemented with 0.25 and 0.50 mM KH_2_PO_3_, No P (-Pi + -Phi) and 1.25 mM H_2_PO_4_^3–^ as the control. Individual cuttings, which had two auxiliary buds, were subcultured in an ethanol-sterilized chamber with laminar air flow and grown in the final treatment media in sterilized glass tubes. Each glass tube contained four identical cuttings of explants. The glass tubes containing the control and experimental units were sealed with the original lids and kept in a culture storage room under constant conditions (16/8 h light/dark cycle, 22 °C temperature, 55–60% relative humidity) for a growth period of 20 days. After 20 days of culture, various physiological traits, such as root length, shoot length, the number of roots, and plant height, were measured [40,41].

#### Experimental Design and Treatments 

A 2 × 4 factorial experiment was conducted in the controlled culture storage room in a completely randomized design with 3 biological replicates. Treatments consisted of two potato genotypes (C31 and C80) and three treatments (0.25 and 0.50 mM Phi, No-P) plus a control. Thirty glass tubes per treatment were arranged in four groups per genotype. Each glass tube contained four explants. After 20 days, three glass tubes containing mature plantlets were randomly selected from each treatment and used for physiological measurements. At the same time, the remaining plantlets were immersed in liquid nitrogen and their shoots were immediately preserved at −80 °C for the transcriptome Illumina sequencing study.

### 2.2. Isolation of RNA, Library Construction, and RNA Sequencing

Total RNA was isolated using a modified CTAB technique [42] from two different plant samples, each subjected to four treatments. A total of 3 g of shoot per sample was utilized as the starting material for the RNA sample preparation. The Epicenter Ribo-zeroTM rRNA Removal Kit (Epicenter, San Antonio, TX, USA) was used to extract ribosomal RNA, and the rRNA-free residue was purified by ethanol precipitation. The NEBNext^®^ Ultra^TM^ Directional RNA Library Prep Kit for Illumina^®^ (NEB, Ipswich, MA, USA) was used to prepare sequencing libraries from rRNA-depleted RNA according to the manufacturer’s instructions. Divalent cations were used to fragment NEBNext First Strand Synthesis Reaction Buffer at high temperatures (5X). The first strand of cDNA was prepared using a random hexamer primer and M-MuLV reverse transcriptase (RNaseH-). Then, DNA polymerase I and RNase H were used to synthesize the second strand of cDNA. In the reaction buffer, the dTTP-containing dNTPs were replaced with dUTP. The remaining overhangs were converted to blunt ends by exonuclease/polymerase activity. NEBNext adaptors were ligated to hairpin loop structures in preparation for hybridization after the 3′ ends of the DNA fragments were adenylated. cDNA segments 150–200 bp in length were identified using AMPure XP technology to purify library fragments (Beckman Coulter, Beverly Hills, CA, USA). Before PCR, 3 L of USER (NEB, USA) containing size-selected, adaptor-gelled cDNA was used for 15 min at 37 °C followed by 5 min at 95 °C. PCR was then performed using Phusion high-fidelity DNA polymerase, universal PCR primers, and an Index (X) primer. Finally, the products were purified (using the AMPure XP system) and the quality of the library was determined using the Agilent Bioanalyzer 2100 system.

### 2.3. Sequencing, Reads Mapping, and Differential Expression Analysis Identification

RNA sequencing with library preparation was performed on an Illumina HiSeq 2500/X platform, and 125/150 bp were generated at Biomics Biotech Co., Ltd. (Beijing, China). Gene expression profiles (collected from fragments per kilobase exon per million mapped fragments (FPKM) to compare samples) were used to perform the principal component analysis (PCA) and Pearson correlation in Ref. [43]. The reference genome and gene model annotation files were obtained directly from the genome website (http://spuddb.uga.edu/dm_v6_1_download.shtml), accessed on 12 June 2022. Bowtie v2.0.6, Ben Langmead, MD, USA [44], was used to build the reference genome index, and (TopHat v2.1.1, Cole Trapnell, MD, USA) was used to align paired-end clean reads with the reference genome. In a reference-based approach, the mapped reads from each sample were created using Scripture (beta2) and Cufflinks (v2.2.1) [45]. 

Clean reads were filtered from the raw data after removing low-quality RNA-seq reads, reads consisting only of adaptors, and reads containing 5% unknown nucleotides (Ns) [46]. All left files of the libraries/samples (read1 files) were combined into a single left.fq file, and all right files of the libraries/samples (read2 files) were combined into a large right.fq file. Trinity [47] was used to assemble the transcriptome based on the left.fq and right.fq, with min kmer cov set to 2 by default and all other parameters set to the default values. The functional annotations of the identified transcripts were based on eight public databases, namely the National Center for Biotechnology Information (NCBI) non-redundant (Nr) protein sequences [48], NCBI non-redundant nucleotide sequences (Nt), and protein family (Pfam) [49]; Clusters of Orthologous Groups of proteins (COG) [50]; Eukaryotic Orthologous Groups (KOG) [51]; Swiss-Prot, a protein sequence database that has been manually annotated and reviewed [52], the Kyoto Encyclopedia of Genes and Genomes (KEGG) [53], and the Gene Ontology (GO) [54]. Blast2GO was used to map terms describing molecular activities, biological processes, and cellular components to the expected genes based on the results of Nr BLASTp searches [55]. Custom Perl scripts were used to perform functional classification of keywords COG and GO for all genes [56]. 

Based on the sample treatments, the differential expression analysis was performed for samples in groups, such as Con vs. Phi0.25, Con vs. Phi0.50, and Con vs. No-P of each genotype to identify differentially expressed genes (DEGs) associated with Phi tolerance. The *DESeq2* package in R was used to perform the genetic analysis, which was based on FPKM. *p* values were corrected for the false discovery rate using the differentially expressed gene analyses with stringent criteria of the log2 fold change (log2FC) ≥ 2 and false discovery rate (FDR) with an adjusted *p*-value (padj) < 0.01 [57]. 

### 2.4. Identification of Transcription Factors in the Differentially Expressed Genes

To identify transcription factors (TFs) involved in Phi response in the potato, iTAK software was used in the default state [58]. The HMM search database (version 3.0) was then used to target open reading frames to the TF protein domain [59]. The TF families available in the PlantTF database version 3.0 (http://planttfdb.cbi.pku.edu.cn), accessed on 6 June 2022, [60] were used to define the aligned sequences.

### 2.5. Validation of DEGs by qRT-PCR

Total RNA was extracted from approximately 100 mg of the frozen shoot tissue using the TransZol Up Plus RNA Kit (lot no. M31018) according to the manufacturer’s instructions. RNA quantity and quality were examined using a TGen spectrophotometer (TianGen) using A260 nm/A280 nm and A260 nm/A230 nm ratios.

Eighteen genes were selected for the quantitative real-time polymerase chain reaction (qRT-PCR) to confirm the validity and reliability of the data from the RNA-seq experiment (Supplementary Table S1). We used Tiangen Biotech The High-Capacity cDNA Reverse Transcription Kit (Tiangen Biotech, Beijing, China) to synthesize new cDNA from the treated RNA according to the manufacturer’s guidelines. A NanoDrop 1000 spectrophotometer was used to measure the concentration of cDNA in all samples [47]. For qRT-PCR assays, a Bio-Rad iQ5.0 instrument (Hercules, CA, USA) with a Roche qPCR kit (Basel, Switzerland) was used and heated to 95 degrees Celsius for three minutes. The RT-qPCR assays were performed using the iQ5.0 instrument (Bio-Rad, Hercules, CA, USA) in conjunction with a SYBR Green qPCR kit (Roche, Basel, Switzerland). Heating was performed for 3 min at 95 °C, followed by 40 cycles of 30 s at 95 °C and 30 s at 60 °C. Dissociation curves were used to evaluate the specificity of the amplicon at the end of each run with amplification reactions (20 L) containing 10 L of 2 × SYBR Green Mix, 1 L of cDNA, and 0.25 M of forward and reverse primers. The 2^−∆∆Ct^ technique [61] was used to estimate relative gene expression, which was then normalized against actin. Experiments were performed three times under the same conditions.

### 2.6. Analysis of Data

Gene expression values are expressed as mean ± standard deviation (SD) (*n* = 3) and were analyzed with SPSS statistical software (version 22.0, SPSS Inc, Chicago, IL, USA) using the one-way method ANOVA at a significance level of 5% probability. Differences between means were determined by Duncan’s multiple range test, and GraphPad Prism 8 was used to generate all graphs. 

## 3. Results

### 3.1. Phi Impedes Physiological Growth and Development of the Potato in a Medium without Pi

**Figure 1:**
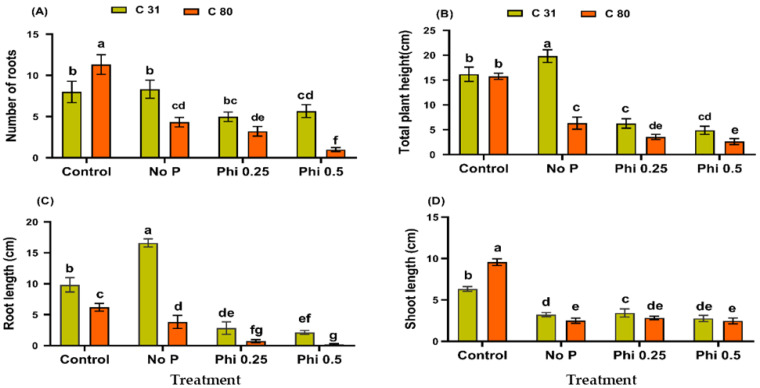
Phi suppressed the physiological growth of two potato genotypes in in vitro propagation for 20 days at different Phi doses. Plants were analyzed for (**A**) difference in the number of roots; (**B**) total plant height (cm); (**C**) root length (cm); and (**D**) shoot length (cm) after 20 days of growth in concentrations of Phi (0, 0.25, 0.5 mM) in the media with or without Pi. Values represent the average of three replicates, and the error bars indicate standard deviation (SD). According to Duncan’s multiple range test (*p* < 0.05), the means with the same lowercase letters are not significantly different from each other.

The effects of Phi on the growth and development of the potato (*S. tuberosum* L.) were studied in vitro using tissue cultures grown in media with or without Pi. Plant growth and development were significantly affected in media containing Phi without Pi. A twofold difference was observed between the two genotypes for all indices measured between Pi-sufficient (control plants) and -deficient plantlets (Phi-treated plants) (Figure 1A–D). However, the presence of 0.50 mM Phi decreased the number of roots of both genotypes (Figure 1A). The reduction in the number of roots was more pronounced in genotype C80 than in C31 compared with the respective control plants. Genotype C31 recorded 70.88% compared to 8.83% in C80 compared to the control plants. At a low Phi concentration of 0.25 mM, a percentage of 62.25% was recorded in C31 and 28.42% in C80. Root length development was significantly reduced by the variation between the two genotypes. Genotype C31 recorded 21.67 and 28.79% versus 2.74 and 11.77% at 0.5 and 0.25 mM Phi, respectively. A similar result was obtained when the effect of Phi on the shoot length was measured. Compared to the control plants, C31 recorded 43.76 and 54.19% versus 9.83 and 25.81% for genotype C80 at 0.50 and 0.25 mM Phi, respectively. Similar suppression of growth was observed at both concentrations (0.50 and 0.25 mM) of Phi in the total plant height of the two genotypes. Consequently, the significant reduction in the number of roots, root lengths, and shoot lengths affected the propagation of the physio-morphological appearances of the plants.

Plant height of the two genotypes was significantly affected compared to their respective controls, but more so in C80 than in C31. Genotype C31 recorded a 69.39 and 61.23% decrease in plant height at 0.50 and 0.25 mM Phi, respectively, compared to genotype C80 (93.32 and 77.63%). In addition, the two genotypes were studied in a medium without Pi and Phi, i.e., No-P. The effect of No-P treatment resulted in a significant decrease in genotype C80 for all measured indices, while genotype C31 showed an increase in all measured indices except shoot length. Genotype C31 showed an increase in the number of roots (4.125%), root length (68.87%), and plant height (22.63%). However, shoot length decreased by 48.97% in C31 compared to the control plant. On the other hand, a reduction in the number of roots (61.78%), root length (38.77%), shoot length (73.88%), and plant height (59.86%) was observed in C80 compared to the control plant.

### 3.2. RNA Sample Sequencing, Quality Control, and Functional Annotation

**Figure 2:**
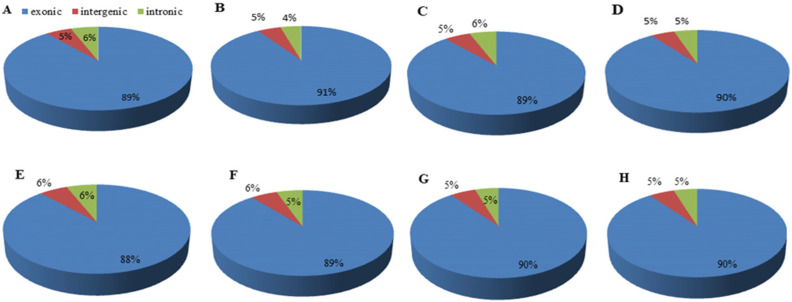
Alignment of transcriptome sequencing reads. (**A**), C31 (control) (**B**); C31 (No-P) (**C**), C31 (Phi0.25); (**D**), C31 (Phi0.5); (**E**), C80 (control) (**F**), C80 (No-P); (**G**), C80 (Phi0.25), and (**H**), C80 (Phi0.5). All treatments were aligned with 3 biological replicates each. Control = 1.25 mM Pi; No-P = (-Phi + -Pi); Phi 0.25 = Phi 0.25 mM Phi or Phi 0.5 = Phi 0.5 mM.

To elucidate the molecular mechanisms underlying increased phosphite tolerance and the genes regulating expression in potato genotypes, we compared the global gene expression profiles of two potato genotypes, C31, which showed some degree of moderate tolerance to Phi in our preliminary studies, and C80, which was susceptible to Phi. RNA samples were collected from two different 20-day-old plants grown in MS media with Phi in two different treatments—the plant without phosphate and the control with 1.25 mM Pi. The average raw reads obtained from C80-Phi 0.25, C80-No-P, C80-Con, C80-Phi 0.50, C31-Phi 0.50, C31-No-P, C31-Con, and C31-Phi 0.25 samples were 39,716,517, 41,101,662, 41,401,177, 41,855,121, 41,941,310, 42,043,470, 42,225,842, and 42,635,159, respectively (Table 1). Of these, the total mapped reads ranged from 27,377,396 bp (in C80 Phi-0.25) to 29,718,175 bp (in C31 Phi-0.25) (Table 1). Of the total mapped reads, more than 99% (≥27,303,171 bp) were uniquely mapped (Table 1). These libraries had guanine–cytosine (GC) content ranging from 41.38 % (in C31-Con) to 42.22% (C31No-P), while the quality control (Q) at 30 error rates (QC30) ranged from 94.46% (in C80No-P) to 94.95% (C31-Con). At least 91% of the clean reads were assigned to the potato reference genome, including at least 89% of the coding region (exon) in C31 (control), C31 (Phi 0.25), or C80 (No-P) (Figure 2A–H). High Pearson correlation coefficients (>0.74) were obtained between samples (Supplementary Figure S1). Together with the successful annotations of most detected genes in public databases, high reproducibility, and repeatability of samples from the same treatment, these results suggest that our transcriptome results are reliable for further downstream analyses. 

#### Gene Annotation and Functional Classification of DEGs

**Figure 3:**
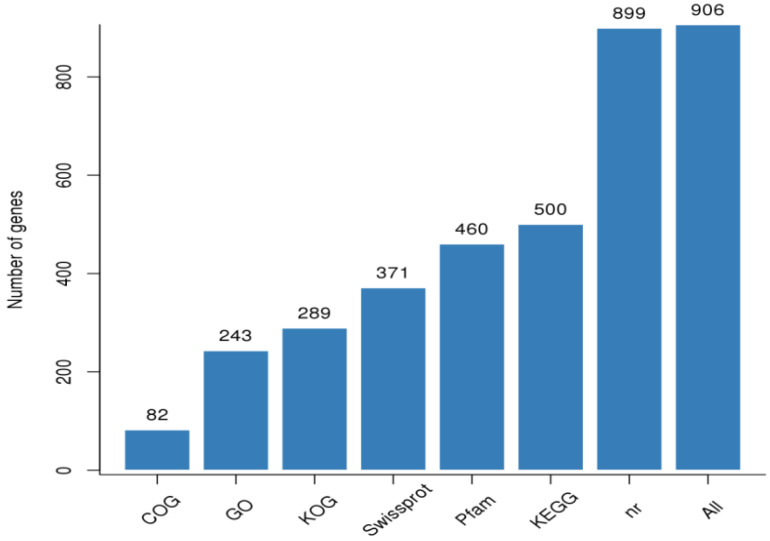
Functional annotations of genes in public databases. All represent the set of annotated genes in at least one of the public databases listed below: NR, non-redundant nucleotide sequences; KEGG, Kyoto Encyclopedia of Genes and Genomes; Swiss-Prot, Swiss Protein; Pfam, Protein Families; GO, Gene Ontology; KOG, Eukaryotic Orthologous Groups; and COG, Cluster Orthologous Groups of Proteins. The number of genes annotated in each public database is shown on top of each bar.

**Figure 4:**
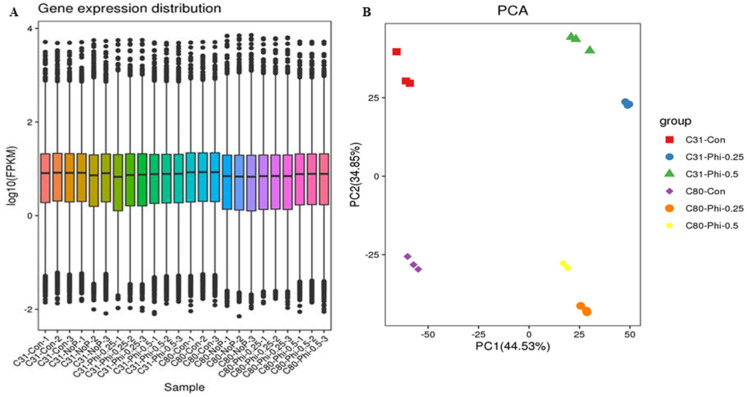
(**A**) Distribution of fragments per kilobase of exon per million mapped fragments (FPKM) in samples. (**B**) FPKM-based principal component analysis of samples. C31 (con); C31 (Phi0.25); C31 (Phi0.50); C80 (con); C80 (Phi0.25); C80 (Phi0.5); each with three samples.

A total of 224,163 genes discovered in the libraries were annotated in at least one of the eight publicly available databases. Of the annotated unigenes, 11,192; 19,423; 23,720; 17,963; 27,163; 26,492; 32,703; and 33,229 were annotated using COG; GO; KEEG; KOG; Pfam; Swiss-Prot; TrEMBL; NCBI nr; and all databases, respectively (Figure 3). Figure 4A shows a high-quality distribution of FPKM with a correlation of samples centered on FPKM that could be replicated among Phi treatments. The FPKM values of the samples were also subjected to the principal component analysis (PCA), grouping the biological replicates of each sample, indicating high reproducibility and reliability of results from each replicate. The first two principal components explained 79.38% of the variability between samples with different amounts of Phi and Pi (Figure 4B). 

### 3.3. Identification of Differentially Expressed Genes in Pairwise Comparison between the Six Categories

**Figure 5:**
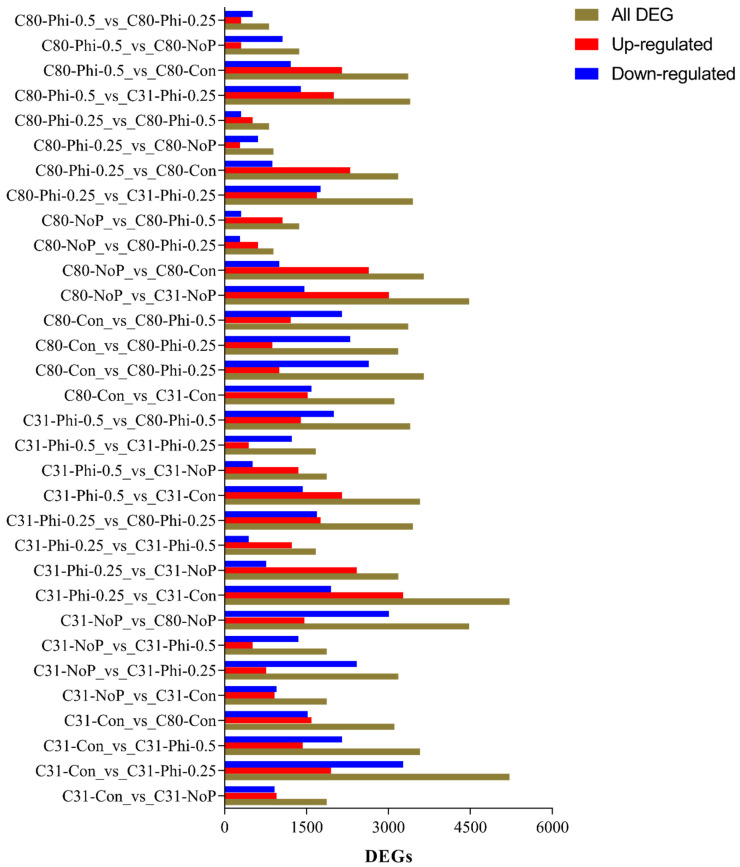
Differentially expressed genes (DEGs) between two potato (*S. tuberosum* L.) genotypes under four different phosphorus conditions (Con, No-P, Phi-0.25, and Phi-0.50 mM KH_2_PO_3_). The brown, red, and blue bars represent the total numbers of up- and downregulated DEGs, respectively. Potato genotype C31 showed some tolerance to Phi, while C80 was susceptible to Phi under a 20-day Phi treatment. Con (phosphate at a concentration of 1.25 mM was used to prepare the media); No-P (no addition of Pi or Phi to the media); Phi 0.25 mM (phosphite at a concentration of 0.25 was used to prepare the media); and Phi 0.50 mM (phosphite at a concentration of 0.50 was used to prepare the media). All analyses were conducted in triplicate.

**Figure 6:**
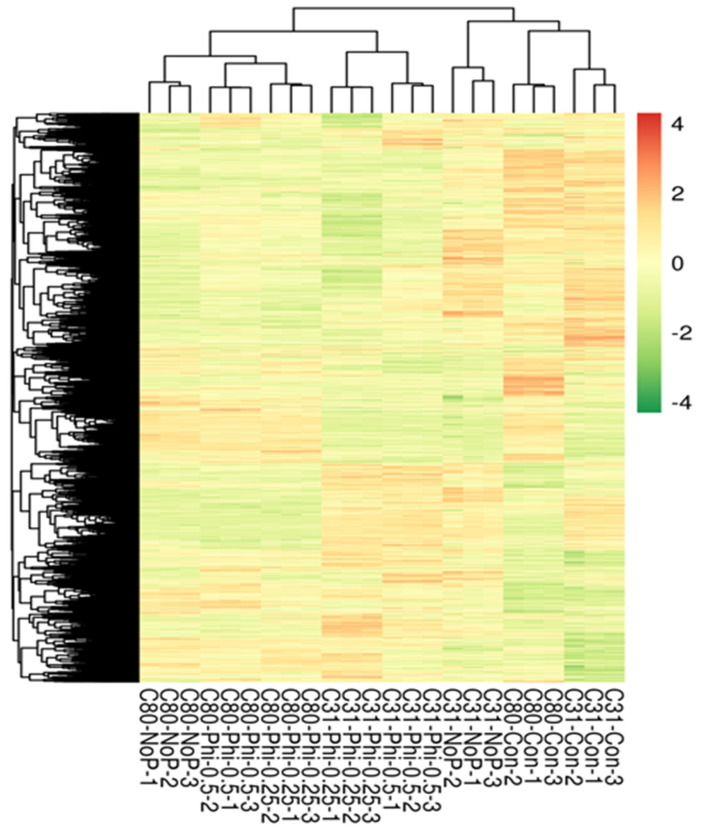
Heatmap clustering of differentially expressed genes (DEGs) with potato genotypes C31 and C80 using RNA-seq expression profiles. C31 (Con, No-P, Phi0.25, Phi0.50) and C80 (Con, No-P, Phi0.25, Phi0.50), after 20 days of therapy with Phi, Pi, and No-P. The color scale of the heat map corresponds to the log2 (FPKM) values of the genes in each sample.

Using the strict threshold, a minimum number of 819 DEGs was detected in both C80-Phi-0.25_vs_C80-Phi-0.50 (consisting of 517 upregulated and 302 downregulated) and C80-Phi-0.5_vs_C80-Phi-0.25 (consisting of 302 upregulated and 517 downregulated) and the maximum number of 5214 DEGs in both C31-Con_vs_C31-Phi-0.25 (consisting of 1947 upregulated and 3267 downregulated) and C31-Phi-0.25_vs_C31-Con (consisting of 3267 upregulated and 1947 downregulated) (Figure 5). The FPKM of the DEGs clustered the samples in a similar manner (Figure 6) as the PCA in Figure 4B. 

**Figure 7:**
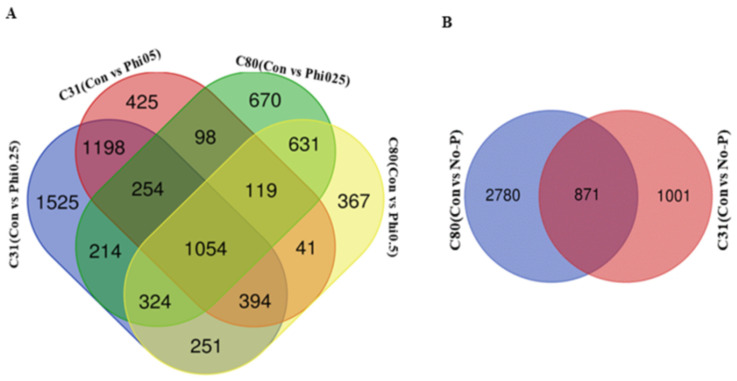
DEGs between four comparisons were analyzed using a Venn diagram; (**A**) C31(Con vs. Phi0.25); C31(Con vs. Phi0.50); C80(Con vs. Phi0.25); C80(Con vs. Phi0.5); and (**B**) C80(Con vs. No-P) and C31(Con vs. No-P), after 20 days of Phi treatment.

In C31 (Con vs. Phi-0.25), 5214 DEGs were found, with 1947 and 3267 up- and downregulated genes, respectively. In C31 (Con vs. Phi-0.50), 3582 DEGs were identified, including 1431 and 2151 up- and downregulated genes, respectively. In C31 (Con vs. No-P), 1872 DEGs were detected, of which, 954 and 918 were up- and downregulated, respectively. In C80 (Con vs. Phi-0.25), 3181 DEGs were detected, of which 877 and 2304 were up- and downregulated, respectively. In C80 (Con vs. Phi-0.50), 3364 DEGs were detected, of which, 1214 and 2149 were up- and downregulated, respectively. In addition, in C80 (Con vs. No-P), 1006 and 2645 were up- and downregulated, respectively, while 3651 DEGs were found. The Venn diagram analysis showed that 1054 DEGs were conserved between the four groups (Figure 7A, Table S2). A total of 871 DEGs were also found between C80 (Con vs. No-P) and C31 (Con vs. No-P) (Figure 7B). The high number of conserved DEGs in Figure 7A, B indicates that a number of DEGs were involved in the regulation of the two genotypes under the different conditions of P. 

### 3.4. 6 DEGs in the Context of Transcription Factors

When both C31 and C80 potato genotypes were subjected to 20 days of Phi treatment, gene expression was either increased or decreased in the major families of transcription factors associated with stress: AP2/ERF (ethylene response factor), Aux/IAA (auxin/indole acetic acid) [62], bHLH (basic helix–loop–helix), bZIP (basic zinc zipper domain), C3H (cysteine3Histidine), MAD (Mother against Dpp), MYB (myeloblastosis), NAC (NAM, ATAF and CUC) [63], PHD (chloroplast envelope-bound plant homeodomain) and WRKY [64] (Table S3). However, we focused on bHLH, MAD, MYB, NAC, and WRKY DEGs to identify potential candidate genes that might be responsible for tolerance or susceptibility to P treatment in the C31 and C80 genotypes, respectively. For this purpose, the conserved DEGs were compared with the four focused TF families under Con, Phi-0.25, Phi-0.50, and No-P, and their FKPM values (log2-transformed) were heat-mapped. 

**Figure 8:**
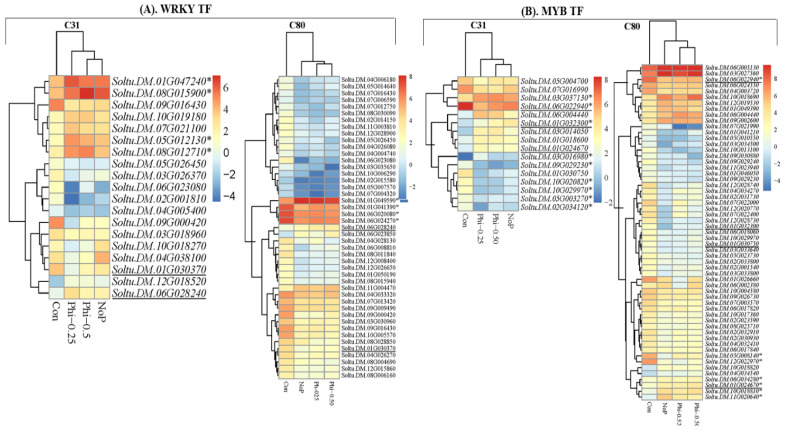
Heatmap of fragments per kilobase exon per million mapped fragments (log2 transformed) conserved transcription factor (TF) genes in either C31 or C80 genotype under four P conditions (Con, Phi-0.25, Phi-0.50, and NoP). (**A**). WRKY TF (**B**). MYB TF. The common conserved genes detected in each TF family are underlined, while those with asterisks (*) are potential candidate genes for tolerance and susceptibility to P starvation observed in C31 and C80, respectively.

A number of WRKY TF genes have been shown to modulate the response of plants to a Pi sufficient amount of Pi, including WRKY33 in Arabidopsis [65], *CaWRKY58* in pepper [66], *GmWRKY46* in soybean [67], and others [68]. A number of WRKY TF genes were detected in the two contrasting genotypes under the four P conditions. Among the numerous WRKY TF genes identified, based on FPKM (log2 transformed), *Soltu.DM.01G047240*, *Soltu.DM.08G015900*, *Soltu.DM.06G012130*, and *Soltu.DM.08G012710* exhibited increased expressions under P deficiency conditions (Phi-0.25, Phi-0.50, and No-P) compared with the control (P sufficiency) in C31 (Figure 8A), indicating their possible involvement in the improvement of P deficiency tolerance observed in C31. In contrast, *Soltu.DM.01G049590*, *Soltu.DM.01G041390*, *Soltu.DM.06G020080,* and *Soltu.DM.06G0*2470 had almost similar expressions under both P sufficiency and P deficiency conditions in the C80 genotype (Figure 8A), indicating their role in enhancing the susceptibility of C80 to P deficiency or Phi. 

MYB TF family is reported to regulate Pi starvation responses either positively or negatively [69]. For example, overexpression of *OsMYB2P*-1 in Arabidopsis and rice increased tolerance to Pi starvation, while the knockdown of *OsMYB2P*-1 by RNA interference in rice made transgenic rice more sensitive to Pi deficiency [69]. A total 51 MYB genes were detected in either the C31 or C80 genotypes under four P conditions (Con, Phi-0.25, Phi-0.50, and No-P). Nineteen and thirty-eight MYB TF genes were detected as conserved genes in C31 and C80, respectively, under four conditions (Figure 8B). *Soltu.DM.03G037130*, *Soltu.DM.06G022940*, *Soltu.DM.01G032300*, *Soltu.DM.03G016980*, *Soltu.DM.09G029230*, *Soltu.DM.10G020820*, *Soltu.DM.10G29970*, *Soltu.DM.05G003270*, and *Soltu.DM.02G034120* exhibited different expression profiles under P sufficiency and deficiency (Figure 8B). These MYB TF genes could be either positive or negative regulatory factors for the P tolerance observed in the C31 genotype. Conversely, five MYB TF genes (*Soltu.DM.05G008140*, *Soltu.DM.12G022970*, *Soltu.DM.06G034280*, *Soltu.DM.01G024670*, and *Soltu.DM.11G020640*) largely under P sufficiency and deficiency conditions (Figure 8B), highlighting their potential roles in the P susceptibility observed in the C80 genotype. 

Other potential candidate genes from bHLH, MAD, and NAC from the C31 and C80 genotypes are shown in Supplementary Figures S2 and S3. *bHLH32* was identified as a negative regulator of Pi deficiency in Arabidopsis in the study by Chutia et al. [70]. MAD box genes have been reported to regulate root hair length and spacing under P availability or absence [71]. NAC genes have been shown to respond to various aspects of nutrient abundance or deficiency, including P [72]. Therefore, the genes reported here with the different TFs could be useful genomic resources for future functional validation studies to ascertain their role in regulating P response in the potato. 

### 3.5. Analysis of COG Enhancement and KEGG Pathway DEGs 

**Figure 9:**
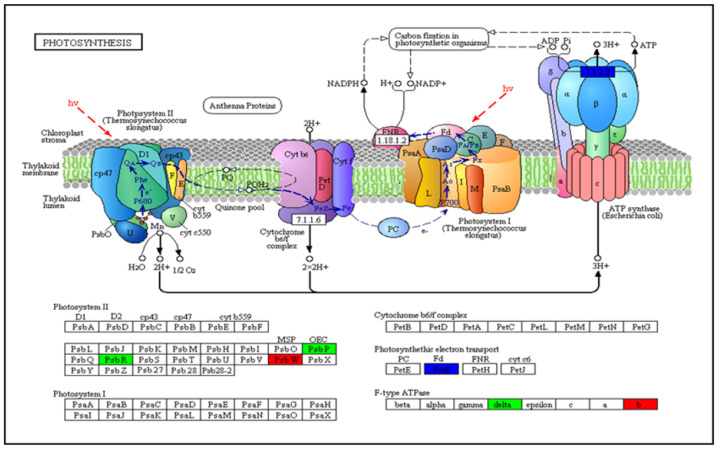
DEG evaluation of photosynthesis in the potato genotype C31 after 20-day treatment with phosphite. Red, green, and blue colors indicate up-, down-, and mixed regulatory forms of DEGs, respectively. After 20 days of growth, each treatment (for both genotypes) was harvested in triplicate for sequencing under P deficiency (0.25, 0.50, and No-P) and P sufficiency (Pi-1.25).

Annotation with the Clusters of Orthologous Groups of Proteins (COG) database revealed that many DEGs in each comparison were not accurately annotated and were therefore assigned to the “general function prediction only” cluster. Based on the number of annotated genes in each group, “transcription”, “replication, recombination, and repair”, “biosynthesis, transport, and catabolism of secondary metabolites”, “signal transduction mechanisms”, “posttranslational modification, protein turnover, chaperones”, and “carbohydrate transport and metabolism” formed the top six DEG groups in C31(Con vs. Phi 0.50) (Supplementary Figure S4A). In addition, “transcription”, “signal transduction mechanisms”, “carbohydrate transport and metabolism”, “biosynthesis, transport, and catabolism of secondary metabolites”, and “replication, recombination, and repair” were the six most important classes in the comparison between C80(Con vs. Phi0.5) (Supplementary Figure S4C). These findings suggest that DEGs in these categories play a vital role in abiotic stress responses in potato genotypes exposed to phosphite for 20 days. To identify the biochemical pathways associated with DEGs, the KEGG was searched for identifiers. The 50 most prominent KEGG pathways allied with DEGs from the C31(Con vs. Phi 0.50) and C80(Con vs. Phi0.5) comparisons are shown in (Supplementary Figure S4B,D), respectively. Pathways that contained the most DEGs included “plant–pathogen interaction”, starch and sucrose metabolism, phenylpropanoid biosynthesis, and plant hormone signal transduction, among others in the two potato genotypes. In addition, many DEGs from the different Phi treatments of the comparison of genotypes C31 and C80 were enriched in the KEGG pathways “photosynthetic antenna proteins”, “plant hormone signal transduction”, starch and sucrose metabolism, and “photosynthesis” with lower enrichment factors and higher Q values (Supplementary Figure S5A,B). In the photosynthesis pathway, five DEGs were found when comparing C31 and C80 genotypes, with three genes downregulated and two upregulated (Figure 9).

### 3.6. Genes Involved in Hormone Signaling in Plants Have Been Discovered for Differential Expression Analysis

**Figure 10:**
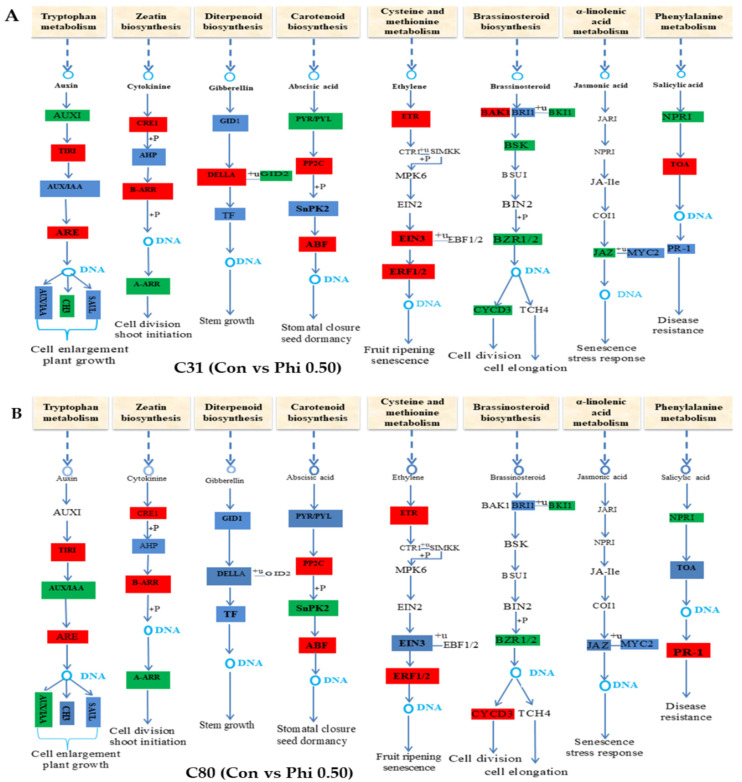
DEGs associated with plant hormone signaling were examined when comparing C31 and C80 genotypes after phosphite treatment. (**A**). C31(Con vs. Phi0.50) (**B**). C80(Con vs. Phi0.50). The colors red and green represent DEGs that are upregulated and downregulated, respectively. DEGs with different regulatory properties are highlighted in blue. After 20 days of growth, each treatment (for both genotypes) was harvested in triplicate for sequencing under P deficiency (0.25, 0.50, and No-P) and P sufficiency (Pi-1.25).

Plants, such as the potato, respond to abiotic stress through a diverse harmonization of various plant hormone pathways, which include auxin (AUX), abscisic acid (ABA), brassinosteroid (BR), cytokinins (CTK), ethylene (ETH), gibberellin (GA), jasmonic acid (JA), and salicylic acid (SA) [73]. A total of 44 genes were found in tryptophan metabolism, of which 21 and 23 were associated with transport inhibitor response 1 (TIR1), auxin response factor (ARF), AUX/indoleacetic acid (IAA)-induced protein (AUX1/IAA), and Auxin in responsive glycoside hydrolase 3 (GH3) were either up- or downregulated in C31 and C80 genotypes (Figure 10A,B, Supplementary Table S4, and Figures S6 and S7). Most of these genes were more highly expressed in Phi0.50 than in Phi0.25 and No-P in both genotypes. For example, the gene *Trans_newGene_199* (TIR1) was increased 1.29- and 1.17-fold in Phi0.50 C31 and C80, respectively. However, at Phi0.25 and No-P, it was not expressed in either genotype. In addition, *Soltu.DM.05G019150* (ARF) showed a 1.17-fold change at Phi0.50 C31, whereas *Soltu.DM.01G035200*; *Soltu.DM.04G036350*; *Soltu.DM.08G029550*; and *Soltu.DM.11G022570* (ARF) showed 1.10; 1.77; 1.69; and 1.21-fold changes in the Phi0.50 C80 genotype. The same gene (ARF) showed 1.09- and 1.01-fold changes in Phi0.25 and No-P only in the C80 genotype, respectively. However, AUX1 and GH3-related genes were downregulated in both genotypes. The results of the tryptophan metabolic pathway show that Phi0.50 induced the TIR1 genes in the C31 genotype, whereas the ARF genes were induced in the C80 genotype.

A total of 22 genes were discovered in zeatin biosynthesis. Of these, 10 were upregulated and 12 downregulated in both genotypes. Phi0.50 induced 2 (B-ARR) genes in C31, one each of CRE1 and B-ARR in C80. Phi0.25 induced *Soltu.DM.08G014610* histidine phosphotransferase (AHP), which was altered 2.66-fold in genotype C31. In addition, Phi0.25 induced *Soltu.DM.01G037840* and *Soltu.DM.11G023770* genes with 1.36-fold and 1.71-fold changes, respectively, and *Soltu.DM.04G003490* (CRE1) was altered 1.42-fold in genotype C80. In addition, *Soltu.DM.05G002670* and *Soltu.DM.07G020230* (B-ARR) were altered 2.28- and 1.54-fold, respectively, in genotype C80.

In diterpenoid biosynthesis, 11 genes related to (DELLA) and gibberellin Insensitive Dwarf 2 (GID 2) were identified in both genotypes. Most genes *DELLA* were expressed in Phi0.50 C31 and delivered in No-P, and Phi025 was expressed from C80. Phi0.50 significantly induced the expression of DELLA genes in C31, while GID2 genes induced strong expression in the C80 genotype.

In carotenoid biosynthesis, most genes (86) were associated with protein phosphatase 2C (PP2C), ABA reactive element binding factor (ABF), pyrabactin resistance 1 (PYR)/PYR -like (PYL) (PYR /PYL), and serine/threonine protein kinase (SnRK2). Of these, 17 PYR /PYL genes expressed in C31 and 10 SnRK2 genes expressed in C80 were downregulated. Fifty-nine genes related to PP2C and ABF were upregulated and highly expressed in the two genotypes relative to the different treatments. However, most of these genes were strongly expressed in Phi0.5, followed by Phi0.25 and No-P. Among the PP2C genes, *Soltu.DM.03G012480* caused a 3.77-, 2.93-, and 2.59-fold change in Phi0.5, Phi0.25, and No-P of the C31 genotype, respectively. While *Soltu.DM.03G022710* (PP2C) caused a 4.38-, 3.52-, and 3.01-fold change in Phi05, Phi0.25, and No-P, respectively, in the C80 genotype. In addition, *Soltu.DM.02G005680* (ABF) caused a 2.01-, 1.83-, 1.34-, and 3.71-, 1.46-, and 3.02-fold change in the C31 and C80 genotypes, respectively. The C80 potato plantlets in the growth media containing Phi and No-P showed yellowed leaves and stunted growth compared to the C31 plantlets. This suggests that plants under Phi stress convert chlorophyll in their leaves to carotenoids by forming abundant structural genes for the carotenoid biosynthetic pathway.

In addition, Phi0.50 induced 12 genes involved in cysteine and methionine metabolism (ETR, ethylene-insensitive protein 3 (EIN3), and ERF1/2), 7 of which were strongly expressed. Three genes were induced by Phi025, whereas No-P induced two genes in both genotypes. Similar results were obtained for brassinosteroid biosynthesis in the two genotypes. *Soltu.DM.01G007270* (BAK1) was expressed at Phi0.25 and No-P in the C31 genotype, whereas *Soltu.DM.02G028240* (CYCD3) was expressed in the C80 genotype. However, no expression was observed for Phi 0.25 and No-P in the C80 genotype. Similarly, 10 TGA genes (4, 5, and 1) were more highly expressed at Phi0.50, Phi0.25, and No-P in the C31 genotype, whereas they were not expressed in the C80 genotype. For example, the gene *Soltu.DM.10G022600* (TGA) showed higher fold changes (by 2.39, 3.06, and 2.18) compared to the other 10 genes in the treatments of the C31 genotype. Again, only the gene *Soltu.DM.07G011890* (NPR1) was expressed in Phi0.25 of C31. In addition, 11 Pathogenesis-Related Protein 1 (PR-1) genes related to phenylalanine metabolism were more highly expressed in C31 than in C80. Thus, 8 of the 11 genes were expressed in C31, whereas 3 genes were expressed in the C80 genotype. This suggests that the phenotypic growth of C31 plants is better than that of C80 plants during their growth period, which may be due to the structure of the α-linoleic acid and phenylalanine genes. 

### 3.7. Expression Patterns of 18 Different Genes in Response to Phi Stress by qRT-PCR

Eighteen genes that were differentially expressed between genotypes C31 and C80 under the four P conditions were randomly selected for qRT-PCR (Supplementary Table S1 and Figure S8). The gene expression response differed between the Phi treatments in the two genotypes. The expression patterns of ten genes (*Soult.DM.12G004810*; *Soltu.DM.03G019440*; *Soltu.DM.02G024820*; *Soltu.DM.03G036150*; *Soltu.DM.01G035900*; *Soltu.DM.02G002360*; *Soltu.DM.09G020830*; *Soult.DM.12G004810*; *Soltu.DM.12G026570*; and *Soltu.DM.12G026290*) were significantly increased in genotype C31 compared with genotype C80 (*p* < 0.05). Of these, four genes (*Soltu.DM.12G004810*; *Soltu.DM.03G019440*; *Soltu.DM.12G026570*; and *Soltu.DM.12G026290*) showed statistically significant expression patterns with 2.2; 2.5; 4.4; and 3.6 higher values compared to the control at 0.50 mMPhi. In addition, five genes (*Soltu.DM.02G024820*; *Soltu.DM.03G036150*; *Soltu.DM.01G035900*; *Soltu.DM.12G026570*; and *Soltu.DM.12G026290*) showed significantly higher expression at 0.25 mMPhi compared with the control. In the case of No-P supply, 3 genes (*Soltu.DM.09G020830*; *Soltu.DM.12G026570*; and *Soltu.DM.12G026290*) showed higher expressions compared with the control. However, the expression patterns of the C80 genotype genes were very low. Three genes (*Soltu.DM.02G002360*; *Soltu.DM.12G026570*; and *Soltu.DM.12G026290*) showed higher expressions at both 0.50 and 0.25 mM Phi, whereas one gene *Soult.DM.01G013680* was more highly expressed in the No-P supply compared with the respective control. The expression profiles were consistent with the results of the RNA-seq data, indicating the reliability of our results. 

## 4. Discussion

In the present study, we investigated the effects of Phi, a phosphate (Pi) analog, on plant growth and the responses of two potato genotypes to Pi deficiency. We found that phosphite at concentrations of 0.25 and 0.5 mM significantly reduced plant growth of the two genotypes in all measured morphological traits compared with the respective control plants. When comparing the inhibitory effects of Phi on the two potato genotypes, the data showed that genotype C31 responded better to Phi than genotype C80 (Figure 1A–D). This result is consistent with our previous Phi screening study conducted with two hundred potato genotypes. In addition, the two potato genotypes were evaluated with a No-P treatment. There was a significant difference between C31 and C80 in the root number, root length, shoot length, and plant height. The inhibitory effect of Phi was observed in the potato [74], tomato [75], and *Brassica* sp. [11]. Although Phi cannot be a substitute for Pi to meet the phosphorus needs of plants, it has recently been shown to be useful in agriculture. In light of this, the mechanisms of tolerance and the components of the signal transduction pathway linking the response to Pi deficiency and Phi sensitivity in the potato need to be investigated.

To understand the dynamics and gain new insights into the molecular basis of the effects of Phi on potato genotypes, the present study compared a global transcriptional profile of C31 and C80 using RNA-seq technology. RNA-seq is a highly efficient sequencing tool that allows researchers to study global transcriptional profiles. It is widely used to study molecular responses to biotic and abiotic challenges and to compare transcriptomes under different conditions [76,77,78,79,80]. 

RNA-seq identified 224,163 genes in the samples, of which 20,864 are functionally annotated in public protein databases, such as COG, KOG, GO, KEGG, Swiss-Prot, Pfam, and the NCBI nr database. The global gene expression profiles of C31 and C80 potato genotypes treated with Phi for 20 days differed from those of control plants and the No-P supply, according to RNA-seq data. When comparing the two potato genotypes, 12,174 genes showed significantly different expression levels in the C31 genotype, of which 1947 and 1431 were upregulated, while 3267 and 2151 were downregulated in C31 (Con vs. Phi0.25 and Phi0.5, respectively). In genotype C80, of the 6545 genes, 877 and 1215 were upregulated, whereas 2304 and 2149 were downregulated (Con vs. Phi0.25 and Phi0.50, respectively). When comparing the two genotypes in the No-P treatment, 1872 and 3651 genes were detected in the C31 and C80 genotypes, respectively. Of these, 954 and 918 were up- and downregulated in C31, respectively, while 1006 and 2645 were up- and downregulated in C80 plants, respectively. Interestingly, only 871 DEGs were present in the comparisons between C31(Con vs. No-P) and C80(Con vs. No-P) (Figure 7B). This could be due to the different expression levels of the different DEGs in each genotype in relation to Phi stress, resulting in different genetic regulation patterns between potato genotypes. 

It is becoming increasingly clear that the endocytosis pathway involving the coat protein clathrin functions in plant cells. Plant genomes contain homologs of mammalian clathrin coat proteins, such as clathrin heavy chain, clathrin light chain, adaptor protein (AP)2 and AP180 subunits, and downstream effectors (e.g., epsin, dynamin, auxilin, heat shock cognate 70, and synaptojanin) [81,82,83]. Moreover, electron microscopy has revealed different stages of clathrin-coated vesicle production in different types of plant plasma membranes [82]. They are essential for signal transduction and regulation of numerous plasma membrane activities that are vital to plants. In our current studies, the endocytosis pathway was expressed in both potato genotypes but was found more frequently in genotype C31. In all species, a ribosome is a macromolecule unit that facilitates protein production [84]. Plants are known to utilize multiple ribosomal proteins for other functions. For example, the multipurpose ribosomal protein S3 is a structural and useful factor of the ribosome as well as a DNA base excision repair enzyme [84]. In our present study, the “ribosome” pathway ranked first among the prominent KEGG pathways for DEGs, in genetic information processing, identified by comparing C31(Con vs. Phi0.50):C80(Con vs. Phi0.50); C31 genotypes were significantly higher compared to C80 genotypes under Phi treatments. This suggests that the C31 genotype can cope with Phi stress by regulating ribosomal metabolism and is tolerant to some extent. 

In addition, several studies have shown that plants, as well as a number of important crops, reactivate their starch stores to produce energy, sugars, and compounds that help buffer stress. This is a critical mechanism for plant fitness and has significant implications for plant productivity in harsh environments. In the current study, starch and sucrose metabolism topped the KEEG pathways of DEGs in mentalism. Starch and sucrose metabolism were higher in the C31(Con vs. Phi0.50) combination than in the C80(Con vs. Phi0.50) combination. This suggests that the C31 potato genotypes can accumulate more starch and sucrose metabolic pathways that function better under Phi stress conditions.

Further analyses of DEGs based on KEGG pathway enrichment led to the identification of the major pathway, plant hormone signaling, in the treatment combinations of both genotypes (map04075) (Figure 10). According to Javid et al. [85], phytohormones are chemical messengers that are produced at extremely low concentrations in one region of the plant and are transmitted to other parts of the plant where they play a critical role in regulating the plant’s response to stress. Phytohormones help plants adapt to adverse environmental conditions, such as extreme pH and temperatures, flooding, drought, high salinity, and organic and inorganic pollutants. Hormone signaling networks are attractive candidates for facilitating protective responses due to their complexity and ability to interact with each other [86]. These genes are involved in the transport of phosphorylation (AHP) into the nucleus and in the transfer of phosphate groups to type A or type B response regulators (ARR) that control cytokinin gene expression. Type A ARRs consist only of the receptor domain (D) and a short C-terminal region that could be used to adapt the downstream response by acting as a negative regulator of the original signal transduction pathway. Type B ARRs have both an output and a receiving domain. The C-terminal output region (blue) of a protein localized in the nucleus phosphorylates DNA in a sequence-specific manner, stimulates transcription (red), involves type-A ARRs and CRFs, and regulates the downstream response to cytokinin. 

According to Wilkinson and Davies [87], ABA is responsible for promoting distress and weakness in plants by closing stomata, which restricts cellular development and produces genes associated with causing weakness, e.g., non-yellow coloration 1 (NYCI), stay green (SGR), pheophytinase (PPH), and pheophorbide an oxygenase gene expression (PAO) [88]. In both genotypes, most of the genes identified in the ABA-induced carotenoid biosynthetic pathway (ABF, PP2C, PYR /PYL, and SnPK2) were more highly expressed in Phi-treated plants than in plants grown in No-P media (Figure 10, Figures S5 and S6). The use of endogenous ABA and genes related to ABA leads to the weakening of plant tissues [89,90,91]. Typical circumstantial evidence has shown that PYR/PYL, PP2C, and SnRK2 are the three main components of ABA signaling pathways that mediate the promotion of ABA to cause stress or senescence in plants [92,93]. This confirms the fact that Phi cannot be synthesized by plants, so a higher dose could cause shocks that lead to plant death. However, the gene PYR/PYL was downregulated in genotype C31 in both No-P and Phi treatments compared to the control combinations. This suggests that the C31 genotype may be tolerant to Phi stress. The higher expression of carotenoid-associated genes observed in the treatment combination of C80 genotypes may be due to chlorophyll deficiency.

Another phytohormone that affects plant growth, development, and stress response is ethylene. Ethylene is thought to bind to receptors localized in the endoplasmic reticulum in the absence of the hormone and acts as a negative regulator of ethylene signal transduction. This phytohormone affects tissue abscission and senescence by inducing effective genes while repressing genes that delay abscission or senescence [94]. In these studies, three ethylene-insensitive genes (*EIN3*, *ETR*, and *ERF*1/2) were either upregulated or downregulated. However, this expression was only significantly observed in the Phi combinations (0.50 and 0.25) compared to the Con vs. No-P combinations. For example, overexpression of ethylene-insensitive3 (EIN3) enhances plant abscission and distress, whereas loss of EIN3-Like1, its homolog, slows plant abscission and distress [95]. The EIN3 protein was identified as a positive ethylene signaling promoter that promotes chlorophyll degradation when chlorophyll physically binds to and activates the promoters of non-yellowing coloration 1 (YC1) and pheophorbide a oxygenase (PAO) [96]. Most genes associated with BR, SA, and JA were upregulated in combinations of control and high Phi compared to control and No-P. These identified genes associated with JA, BR, and SA can be further explored and targeted to improve Phi tolerance in the potato. 

Transcription factors (TFs) are thought to play a critical role in regulating adaptive mechanisms among the molecular drivers of Pi stress responses [93,97,98,99,100]. TFs have been shown to interact with cis-transcriptional regulatory elements surrounding the genes they regulate, such as promoters, silencers, enhancers, insulators, and LCR regions, thereby affecting the expressions of many downstream genes to control a variety of biological processes [23,79,101]. A small change in the relative abundance of transcription factor messenger RNAs can set in motion a chain of events that affect a variety of physiological processes and lead to significant changes in the expression of downstream genes [102]. In the present study, several TF families that regulate gene expression were discovered. The major TFs discovered include: AP2, AUX /IAA, bHLH, bZIP, C3H, MAD, MYB, NAC, PHD, and WRKY. However, we focused on the bHLH, MAD, MYB, NAC, and WRKY DEGs to find potential candidate genes responsible for Phi tolerance or susceptibility in the C31 and C80 genotypes (Figure 8, Supplementary Figures S2 and S3). WRKY transcription factors have been found to play a role in nutrient deficit response signaling pathways in several studies [101,103]. In our current experiment, several genes related to WRKY TF were highly expressed under Phi treatment compared to the corresponding control, Pi-sufficient. This suggests that they are actively involved in enhancing Phi tolerance in the C31 genotype. In C80, the expressions of genes related to WRKY TF were similar in P deficiency (Phi-0.25, -0.50, and No-P) as in P sufficiency, confirming the sensitivity of this genotype to Phi. Jiang et al. [84] showed that *AtWRKY75* is the first WRKY member involved in the regulation of Pi deficiency. Thus, *AtWRKY75* was significantly increased in Pi insufficiency in Arabidopsis plants, and RNAi suppression of *WRKY75* resulted in early anthocyanin buildup, suggesting that RNAi plants are more susceptible to Pi stress [36]. Similar reports suggest that *WRKY6*, *WRKY42*, and *WRKY75* are induced by Pi deficiency [36,104]. According to Yi et al. [37], plant tolerance to Pi deficiency is mediated by transcriptional control of Pi stress response genes by bHLH family members. In addition, *TabHLH1* is sensitive to exogenous Pi deprivation stressors and confers increased Pi tolerance in wheat [7]. MYBs are one of the best-studied transcription factors involved in the response to Pi deficiency [105]. Moreover, stress tolerance in Pi deficiency is controlled by NAC and MAD TFs [72,106]. In particular, the gene *ZmNAC134* has been associated with low Pi tolerance in maize seedlings [107]. In Arabidopsis, rice, and other plant species, MADS genes are also transcriptional responses to Pi deficiency and are important in mediating plant tolerance to Pi deficiency stress [4,108]. qRT-PCR has been shown to be an advantageous tool for validating transcriptomics data due to its precision, accuracy, simplicity, speed, and sensitivity [109]. The genes discovered in this study could be selected for functional validation experiments to decipher and validate their regulatory involvement in Phi stress in potatoes, especially those verified by qRT-PCR. Analysis of the expression of TFs revealed that WRKY, MYB, NAC, and bHLH were largely associated with Phi-tolerant genotype C31, whereas MAD was associated with susceptibility of genotype C80. The results presented in the present study provide the basis for manipulating and understanding the response of phosphite in potatoes to exploit its potential for crop production.

## 5. Conclusions 

In the present study, transcriptome profiling was performed to identify genes with differential expressions in two potato genotypes under different P conditions. This was demonstrated by examining the transcriptional changes induced by the supply of 0.25 and 0.50 mM No-P and the corresponding control. It confirmed that Phi and No-P administration reprogrammed plant hormone signaling, TFs, and metabolic pathways, such as tryptophan, zeatin, carotenoids, cysteine, and methionine, which significantly contributed to the promotion and regulation of the effects of Phi in the two genotypes. The present study confirmed that plants cannot metabolize Phi. Genotype C31 showed some degree of tolerance to the compound in the media. Genes detected in the tryptophan metabolic pathway initiated by auxin (TRJ, ARE, Auxi1, CH3, ARF, and Aux/IAA) were more highly expressed in Phi-0.25 or Phi-0.50 mM than in the No-P and Pi combinations. A similar trend in gene expression was also observed for zeatin biosynthesis and cysteine and methionine metabolism induced by cytokinin and ethylene, respectively. However, most of the genes associated with the metabolic pathways identified in this study that regulate plants under stress were more highly expressed in the C31 genotypes than in the C80 genotypes. An analysis of the expressions of TFs revealed that WRKY, MYB, NAC, and bHLH were largely associated with Phi tolerance in the C31 genotype, whereas MAD was related to susceptibility in the C80 genotype. The results of this study provide valuable information on gene functions and pathway engineering under Phi stress in the potato. Future research could focus on the Phi-tolerant C31 genotype to measure its yield potential under Phi treatment in the field.

## Figures and Tables

**Table 1:** Statistics of sequencing data for the two potato genotypes of RNA-seq libraries.

Samples	Raw Reads (bp)	Reads Mapped (bp)	Unique Mapped (bp)	GC (%)	≥Q30 (%)
C31-Con	42,225,842	29,434,183	29,367,816	41.38	94.95
C31No-P	42,043,470	29,486,431	29,421,504	42.22	94.88
C31Phi0.25	42,635,159	29,718,175	29,643,683	41.98	94.50
C31Phi0.5	41,941,310	29,174,524	29,104,150	41.69	94.77
C80-Con	41,401,177	28,685,887	28,618,083	41.52	94.57
C80No-P	41,101,662	28,165,842	28,087,967	41.78	94.46
C80Phi0.25	39,716,517	27,377,396	27,303,171	42.02	94.48
C80Phi0.5	41,855,121	29,440,480	29,365,884	42.10	94.57

Con (phosphate at a concentration of 1.25 mM was used to prepare the media); No-P (no addition of Pi or Phi to the media); Phi 0.25 mM (phosphite at a concentration of 0.25 was used to prepare the media); and Phi 0.5 mM (phosphite at a concentration of 0.5 was used to prepare the media). The two potato genotypes, C31 and C80, were propagated in each treatment medium with 3 replicates and maintained under tissue culture conditions for 20 days. After 20 days of growth, each treatment was harvested with 3 replicates for sequencing; therefore, the values shown are the averages of the 3 replicates (samples) per treatment.

## Data Availability

Not applicable.

## Supplementary Materials

The following supporting information can be downloaded at: https://www.mdpi.com/article/10.3390/genes13081379/s1, Figure S1: Pearson correlation coefficients (>0.74) between samples; Figure S2: Heatmap of fragments per kilobase exon per million mapped fragments (log2 transformed) conserved NAC transcription factor (TF) genes in either C31 or C80 genotype under four P conditions (Con, Phi-0.25, Phi-0.50, and No-P). (A). C31. (B). C80; Figure S3: Heatmap of fragments per kilobase exon per million mapped fragments (log2 transformed) conserved transcription factor (TF) genes in either C31 or C80 genotype under four P conditions (Con, Phi-0.25, Phi-0.50, and No-P). (A). bHLH TF (B). MAD TF; Figure S4: comparisons with COG and KEGG classifications of DEGs of C31(Con vs. Phi-0.5) (A, B) and C80(Con vs. Ph-0.5) (C, D) grown under phosphite treatment during a 20-day growth period; Figure S5: The twenty most enriched KEGG pathways representing six pairwise groupings based on differentially expressed genes. A, C31(Con vs. Phi-0.5) and B, C80(Con vs. Ph-0.5) after 20-day of Phi treatment; Figure S6: DEGs associated with plant hormones signaling were examined when comparing C31 and C80 genotypes after phosphite treatment. Latter C, represents C31(Con vrs Phi-0.25), while D represents C80(CON vrs Phi-0.25); Figure S7: DEGs associated with plant hormones signaling were examined when comparing C31 and C80 genotypes after phosphite treatment. Latter E, represents C31(Con vrs No-P), while F represents C80(Con vrs No-P); Figure S8: qRT-PCR validation of RNA-seq gene expression results; Table S1: Primer pair of the genes used in this study; Table S2: Differentially expressed genes (DEGs) were compared between the two genotypes and their treatments over the course of 20 days of Phi treatment; Table S3: Summary of differentially expressed genes (DEGs) associated with transcription factor families in C31 and C80 genotypes under Phi and No-P treatments; Table S4: Differentially expressed genes (DEGs) associated with plant hormone signaling in C31 and C80 genotypes after Phi and No-P treatments were classified.
