# Supplementary material for: Comparative Transcriptome Profiling Reveals Potential Candidate Genes, Transcription Factors, and Biosynthetic Pathways for Phosphite Response in Potato (Solanum tuberosum L.)
Source: Genes (Basel). 2022 Aug 1;13(8):1379. doi: 10.3390/genes13081379 (PMC9407107; doi:10.3390/genes13081379)
Supplement: Supplementary file 1 [file genes-13-01379-s001.zip › genes-1833171-SM.pdf]

Supplementary Table S1. Primer pair of the genes used in this study

Gene ID	Sequence (5′ to 3′)	Product Length
Soltu.DM.05G018750-F	TGCAGGTTTTATGGGGGCAA	191
Soltu.DM.05G018750-R	GCCATCCAAGTGCCCATACT	
Soltu.DM.06G024260-F	TTCAGGGGCGACATAAGCTG	176
Soltu.DM.06G024260-R	ATTTCTGCCATAGTCGCGGT	
Soltu.DM.07G027100-F	ACCCCTTGAGTTGTGGCATC	199
Soltu.DM.07G027100-R	CTCAAGCCGATGCTTGGAGA	
Soltu.DM.12G004810-F	TGCCTCACATTTGTACGCCT	178
Soltu.DM.12G004810-R	GCACTCGACAATTGGTGCTC	
Soltu.DM.03G019440-F	TCATGTGGCAAATCGAGGGT	167
Soltu.DM.03G019440-R	CACGATCATTGCCTTGAGCA	
Soltu.DM.05G023730-F	TACTTCGCGCCTGTTGGATT	184
Soltu.DM.05G023730-R	GCTGTAGTAACCGGCGATGA	
Soltu.DM.06G005440-F	TACTTCGCGCCTGTTGGATT	184
Soltu.DM.06G005440-R	GCTGTAGTAACCGGCGATGA	
Soltu.DM.06G022730-F	CTTCCCCATCAGTCCACTCG	150
Soltu.DM.06G022730-R	TCGGGGATGGAATCGGAGAT	
Soltu.DM.01G031540-F	CCGTGACTTTTTGCACCGTT	160
Soltu.DM.01G031540-R	CGTTGATCCTATCGTCGCCA	
Soltu.DM.02G024820-F	AGGGTGACGCCACAACATAG	194
Soltu.DM.02G024820-R	ACCCCATTTGCTGAACAGGT	
Soltu.DM.03G022710-F	CCGTGACTTTTTGCACCGTT	120
Soltu.DM.03G022710-R	CGTTGATCCTATCGTCGCCA	
Soltu.DM.03G036150-F	AGCTCTCCCTGTGCCTTACT	154
Soltu.DM.03G036150-R	CAGCGTTACACCCCCACAAT	
Soltu.DM.01G035900-F	GGTAACTTGTCAGCACGGGA	153
Soltu.DM.01G035900-R	CGTCATTGCTCGAGTTGCAG	
Soltu.DM.02G002360-F	CGAATTGTCGTTTCTGGGCG	198
Soltu.DM.02G002360-R	CTTTTCAGCTGCCCAATGCT	
Soltu.DM.01G013680-F	ACTCGTGGACACCTGCTTTT	**163**
Soltu.DM.01G013680-R	GTCAGCATGGCTAAGGAAGGA	
Soltu.DM.09G020830-F	CGGCGTAGCAATAGTGGGAT	**157**
Soltu.DM.09G020830-**R**	CAGAGCGAGAAGCCCCAATA	
Soltu.DM.12G026570-F	CGGAAGCGGATCCAGGATTT	**172**
Soltu.DM.12G026570-R	ATGAGCAGTGGCGGATGTAG	
Soltu.DM.12G026290-F	CCGGATAAGCTCGGGTTCTG	**167**
Soltu.DM.12G026290-R	CTGCTAAAGCATTCGACGCC	
Actin-F	AGGAGCATCCTGTCCTCCTAA	
Actin-R	CACCATCACCAGAGTCCAACA	

Supplementary Table S2. Differentially expressed genes (DEGs) were compared between the two genotypes and their treatments over the course of 20 days of Phi treatment.

**Set of DEG**	**Total DEGs**	**Up regulated**	**Down regulated**
C31(Con vs Phi 0.25)	5,214	1,947	3,267
C31(Con vs Phi 0.5)	3,582	1,431	2,151
C31(Con vs No-P)	1,872	954	918
C80(Con vs Phi 0.25)	3,181	877	2,304
C80(Con vs Phi 0.5)	3,364	1,215	2,149
C80(Con vs No-P)	3,651	1,006	2,645

Supplementary Table S3. Summary of differentially expressed genes (DEGs) associated with transcription factor families in C31 and C80 genotypes under Phi and No-P treatments.

		C31			C80		
Transcription factor	DEGs	Up regulated	Down regulated	Total	Up regulated	Down regulated	Total
AP2/ERF	26	11	15	26	4	22	26
AUX/IAA	105	49	56	105	32	69	101
bZIP	38	17	21	38	17	17	34
C3H	97	55	42	97	38	42	80
PHD	44	34	10	34	10	26	36

* Up represents up-regulated and Down denotes down-regulated

Supplementary Table S4. Differentially expressed genes (DEGs) associated with plant hormone signalling in C31 and C80 genotypes after Phi and No-P treatments were classified.

			**C31 genotype**		**C80 genotype**	
**Plant hormones**	**Unigenes identified**	**Total DEGs**	**Up**	**Down**	**Up**	**Down**
Auxin signaling	45	19	2	9	4	4
Cytokinin signaling	27	15	3	3	5	4
Gibberellin signaling	14	10	3	2	3	2
Abscisic signaling	81	16	6	3	5	2
Ethylene signaling	27	12	6	1	3	2
Brassinosteroid signaling	21	16	3	8	3	2
Jasmonic acid signaling	37	8	1	3	2	2
Salicylic acid signaling	26	12	6	1	3	2

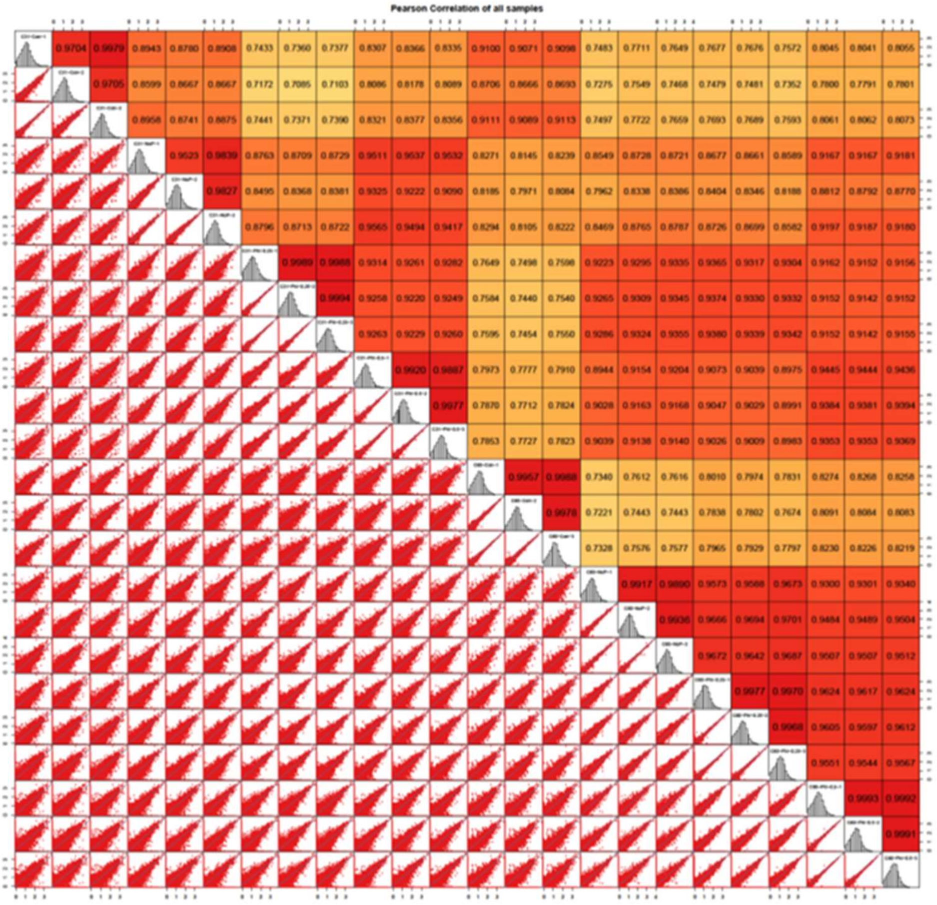

Supplementary Figure S1. Pearson correlation coefficients (> 0.74) between samples. Both potato genotypes C31 and C80 were propagated in each treatment medium with 3 replicates and maintained under tissue culture conditions for 20 days. After 20 days of growth, each treatment for both genotypes were harvested in triplicate for sequencing under P deficiency (0.25, 0.5, and No-P) and P sufficiency (Pi-1.25).

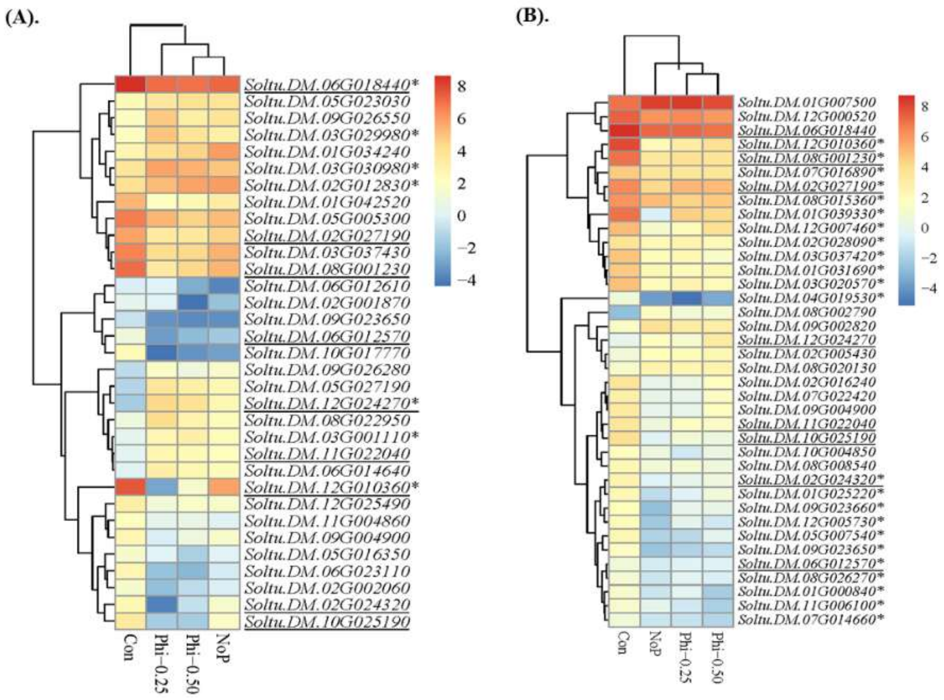

Supplementary Figure S2: Heatmap of fragments per kilobase exon per million mapped fragments (log2 transformed) conserved NAC transcription factor (TF) genes in either C31 or C80 genotype under four P conditions (Con, Phi-0.25, Phi-0.50 and NoP). (A). C31. (B). C80. The common conserved genes detected in the two genotypes are underlined, while those with asterisks (*) are potential candidate genes for tolerance and susceptibility to P starvation observed in C31 and C80, respectively.

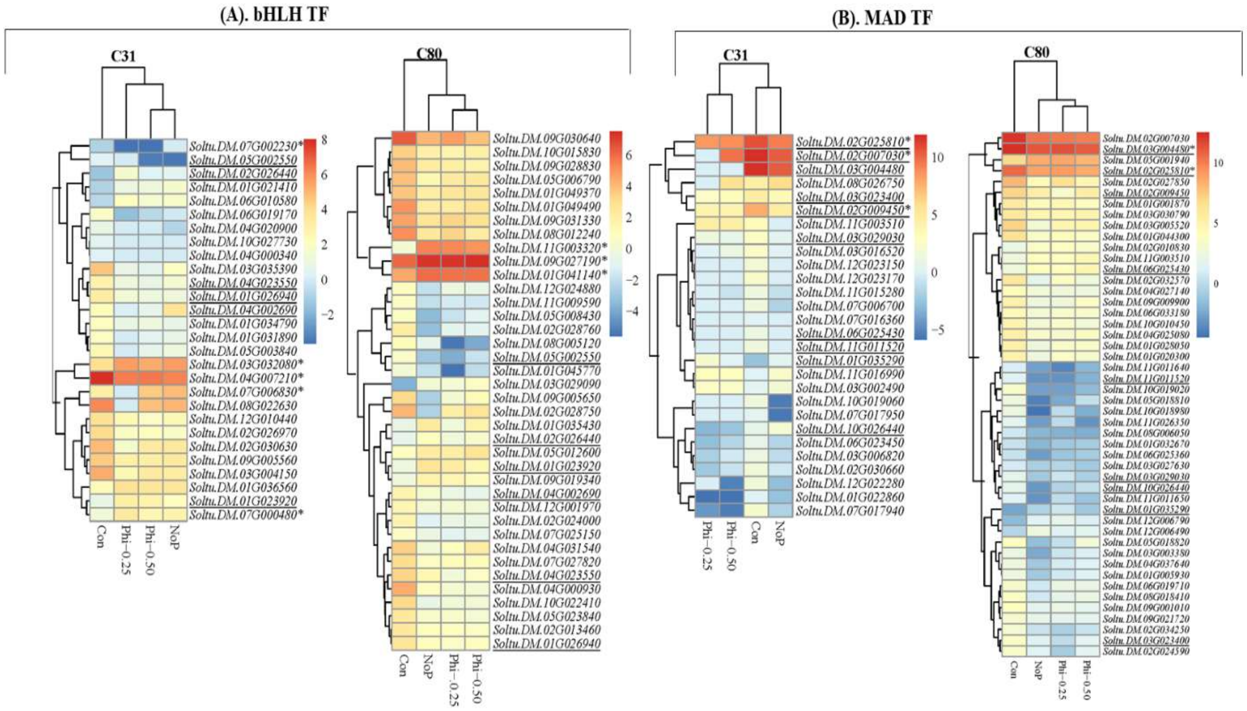

Supplementary Figure S3: Heatmap of fragments per kilobase exon per million mapped fragments (log2 transformed) conserved transcription factor (TF) genes in either C31 or C80 genotype under four P conditions (Con, Phi-0.25, Phi-0.50 and NoP). (A). bHLH TF (B). MAD TF. The common conserved genes detected in each of TF family are underlined, while those with asterisks (*) are potential candidate genes for tolerance and susceptibility to P starvation observed in C31 and C80, respectively.

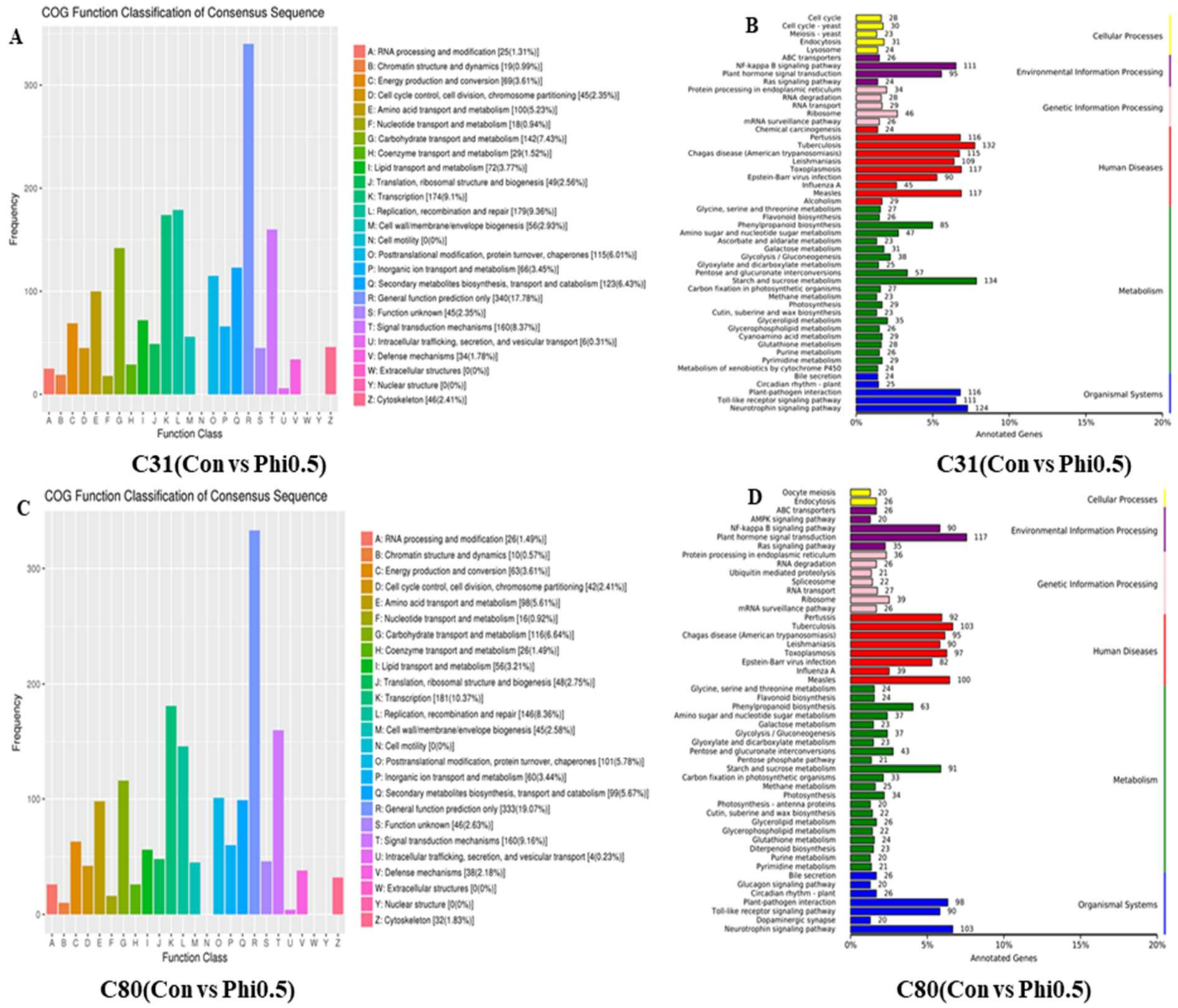

Supplemental Figure S4. comparisons with COG and KEGG classifications of DEGs of C31(Con vs Phi-0.5) (A, B) and C80(Con vs Ph-0.5) (C, D) grown under phosphite treatment during a 20-day growth period. Both potato genotypes C31 and C80 were propagated in each treatment medium with 3 replicates and maintained under tissue culture conditions for 20 days. After 20 days of growth, each treatment for both genotypes were harvested in triplicate for sequencing under P deficiency (0.25, 0.5, and No-P) and P sufficiency (Pi-1.25).

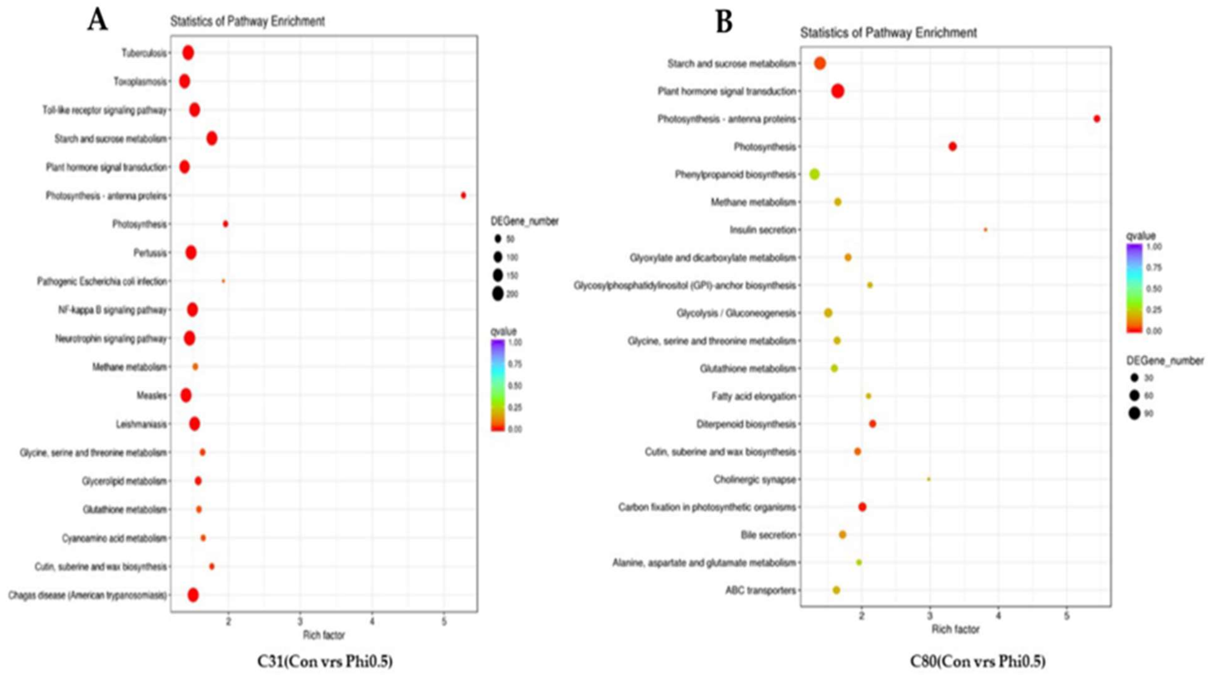

Supplementary Figure S5. The twenty most enriched KEGG pathways representing six pairwise groupings based on differentially expressed genes. A, C31(Con vs Phi-0.5) and B, C80(Con vs Ph-0.5) after 20-day of Phi treatment. After 20 days of growth, each treatment for both genotypes were harvested in triplicate for sequencing under P deficiency (0.25, 0.5, and No-P) and P sufficiency (Pi-1.25).

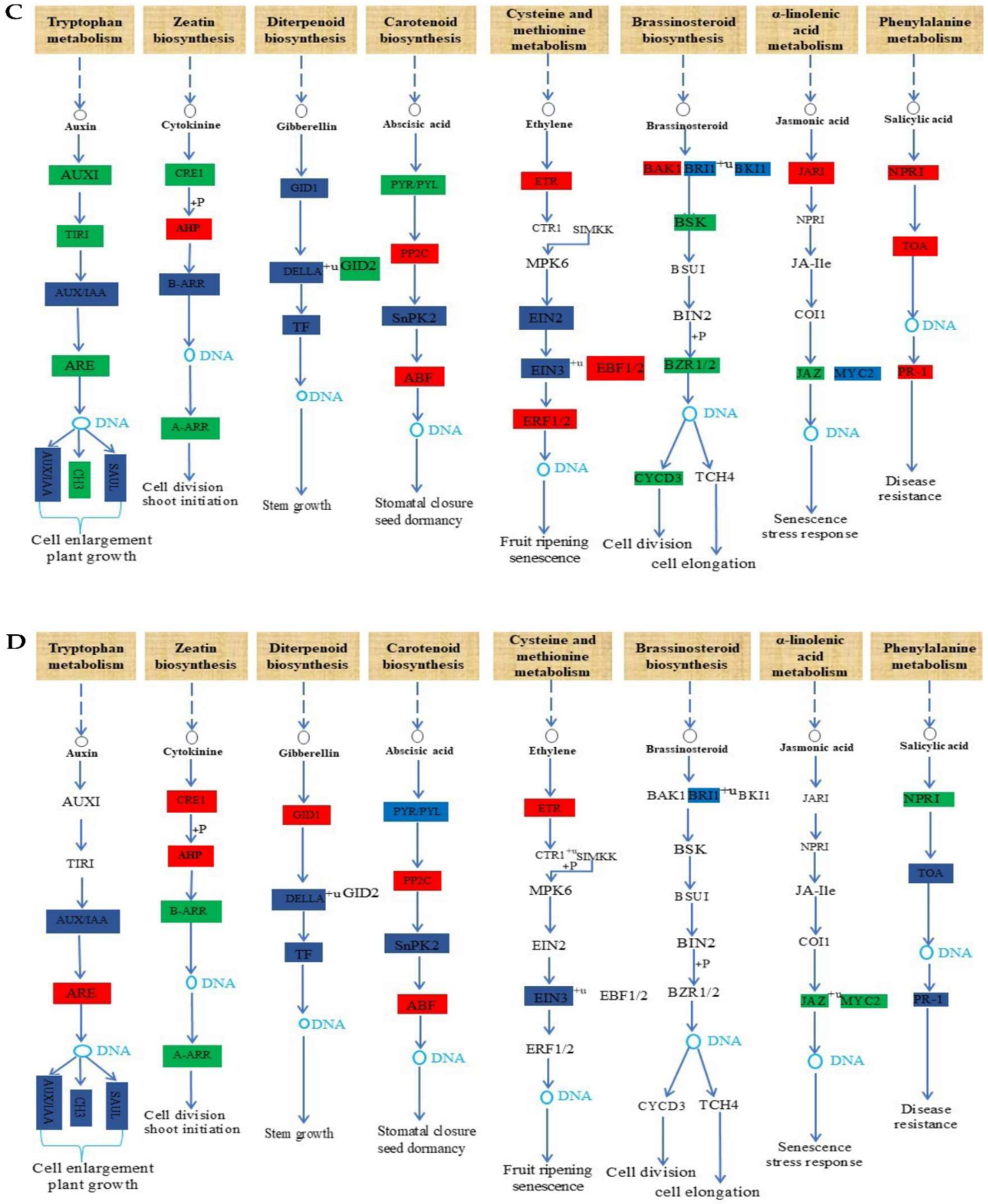

Supplementary Figure S6. DEGs associated with plant hormones signaling were examined when comparing C31 and C80 genotypes after phosphite treatment. Latter **C**, represents C31(Con vrs Phi0.25), while **D** represents C80(CON vrs Phi0.25). The colours red and green represent DEGs that are upregulated and downregulated, respectively. DEGs with different regulatory properties are highlighted in blue. After 20 days of growth, each treatment for both genotypes were harvested in triplicate for sequencing under P deficiency (0.25, 0.5, and No-P) and P sufficiency (Pi-1.25).

E

F

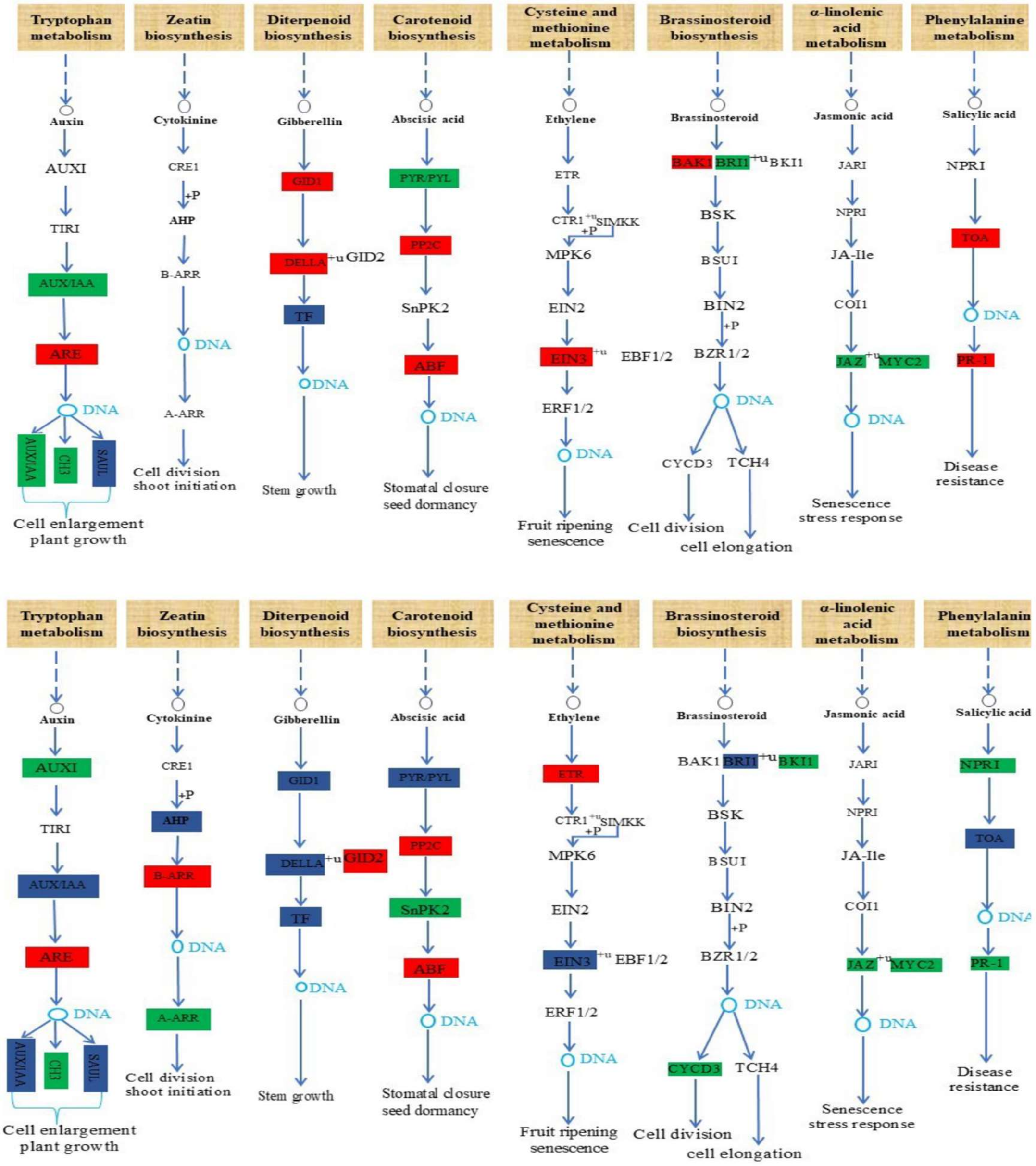

Supplementary Figure S7. DEGs associated with plant hormones signaling were examined when comparing C31 and C80 genotypes after phosphite treatment. Latter **E,** represents C31(Con vrs No-P), while **F** represents C80(Con vrs No-P). The colours red and green represent DEGs that are upregulated and downregulated, respectively. DEGs with different regulatory properties are highlighted in blue. After 20 days of growth, each treatment for both genotypes were harvested in triplicate for sequencing under P deficiency (0.25, 0.5, and No-P) and P sufficiency (Pi-1.25).

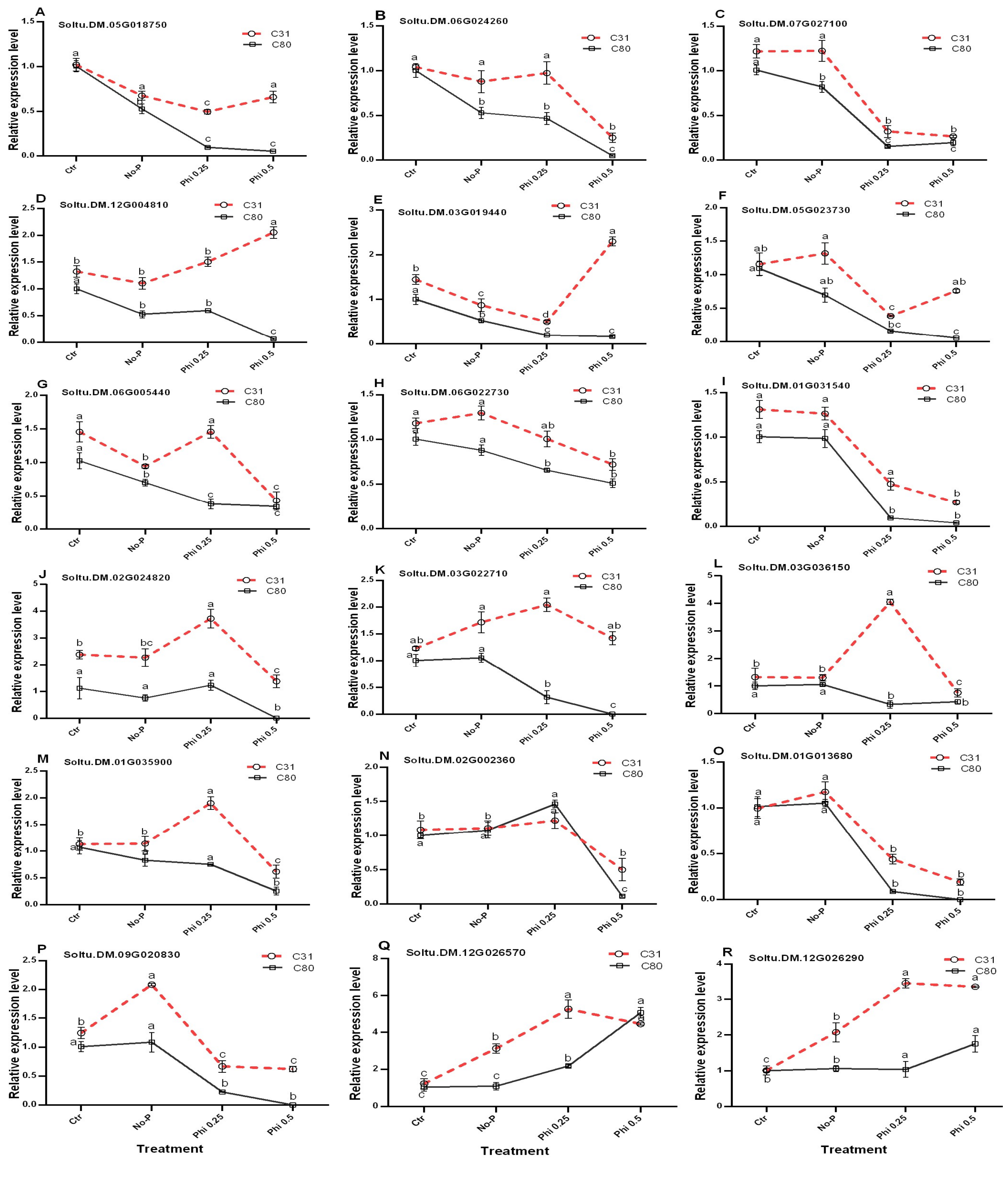

Supplementary Figure S8. qRT-PCR validation of RNA-seq gene expression results. The dashed red lines represent the control, No-P, 0.25 and 0.50 mM for C31; the continuous black lines represent the same treatment for the C80 genotype. The name of the gene appears at the top left of each line graph. After 20 days of growth, each treatment for both genotypes were harvested in triplicate for sequencing under P deficiency (0.25, 0.50, and No-P) and P sufficiency (Pi-1.25). A - *Soltu.DM.05G018750*; B – *Soltu.DM.06G024260*; C - *Soltu.DM.07G027100*; D – *Soltu.DM.12G004810*; E – *Soltu.DM.03G019440*; F - *Soltu.DM.05G023730*; G – *Soltu.DM.06G005440*; H - *Soltu.DM.06G022730*; I - *Soltu.DM.02G024820*; J – *Soltu.DM.01G031540*; K - *Soltu.DM.02G024820*; L – *Soltu.DM.03G022710*; M - *Soltu.DM.03G036150*; N -

Soltu.DM.01G035900; O - *Soltu.DM.02G002360*; P - *Soltu.DM.01G013680*; Q – *Soltu.DM.09G020830*; X – *Soltu.DM.12G026570*; and R - *Soltu.DM.12G026290*.
